# How Does Experience of Violence in Childhood Affect Work Outcomes? A Systematic Review of Longitudinal Studies

**DOI:** 10.1177/15248380251376352

**Published:** 2025-11-28

**Authors:** Clare Tanton, Jodie Pearlman, Katherine Merrill, Ligia Kiss, Agnes Kyamulabi, Louise Knight, Fred Kasalirwe, Russell Burke, Eddy Walakira, Karen Devries

**Affiliations:** 1 London School of Hygiene and Tropical Medicine, UK; 2 University of Illinois Chicago, USA; 3 University College London, UK; 4 Makerere University, Uganda

**Keywords:** child abuse, prevention of child abuse, violence exposure, workplace violence, work

## Abstract

Experiencing violence in childhood and adolescence is both common and long-lasting, and associated with poor short- and long-term health and economic outcomes. In the current study, we reviewed evidence from longitudinal studies on the association between violence in childhood and work outcomes to determine the direction and magnitude of the association, explore variations by violence type, identify evidence gaps, and describe the extent of research and findings on mediators. We systematically searched nine databases for longitudinal studies reporting on the association between violence in childhood and work outcomes, and conducted a narrative synthesis. We identified 46 reports of 27 cohorts, with all but one cohort from high-income countries. This review shows that there is strong evidence from high-income countries that violence in childhood is associated with a range of negative work outcomes. Evidence is strongest for official reports of child abuse and neglect, physical violence, bullying, and composite violence measures, but is more mixed for sexual violence. There is less evidence for emotional violence, witnessing violence, neglect, and adolescent intimate partner violence. Associations are similar for men and women. Nine reports conducted mediation analyses, mainly examining educational factors as mediators. Evidence suggests that educational factors may partially mediate the relationship between violence and negative work outcomes. Cognition, mental health, and noncognitive skills may also be mediators. There is a need for data from low- and middle-income countries, and further mediation analyses to help guide efforts to reduce negative consequences of violence.

## Introduction

Over one billion children and adolescents aged 18 and under report experiencing violence in the past year (Hillis et al., 2016). Patterns of exposure to physical, sexual and emotional violence in childhood are gendered and differ by age and other dimensions, with adolescent girls often, but not always, experiencing more sexual violence versus boys and younger children (Devries et al., 2018; K. M. Devries, Child, et al., 2014; Pinheiro, 2006; Stoltenborgh et al., 2011; Wandera et al., 2017), Children with disabilities experience more violence than non-disabled counterparts (Devries, Kyegombe, et al., 2014; Jones et al., 2012). Violence in childhood is known to have a range of short- and long-term consequences, including poorer mental, physical, and sexual health, increased risk behaviors, and both further victimization and perpetration of violence (K. Devries et al., 2016; Dube et al., 2001; Norman et al., 2012).

Those experiencing violence also have poorer socioeconomic outcomes, including markers of employment such as lower income, unemployment, and job type (Bunting et al., 2018; Fry et al., 2018). However, to date, no systematic review has been specifically designed to examine the association between exposure to specific forms of violence in childhood and adult work outcomes, including income, unemployment, job type, hazardous work, work-related sickness, sex work, or child labor/trafficking. Existing reviews have focused on a narrow range of outcomes and violence exposure (e.g., not including childhood bullying [Wolke & Lereya, 2015]) and have found that associations with work outcomes differed for different forms of violence. There is also a need to explore the relationship between specific exposures and outcomes and how these are influenced by gender, since both experiences of violence and work are highly gendered (Wolke & Lereya, 2015).

Moreover, no reviews have addressed *how* experiences of violence in childhood might affect adult work outcomes. Experience of multiple forms of abuse, particularly within the first decade of life, can lead to developmental delays, including cognitive, language, motor, and socialization skills (Culp et al., 1987). Studies have found negative associations between trauma symptoms and cognitive functioning (Culp et al., 1987; De Bellis et al., 2005), and career development (Coursol et al., 2001; Strauser et al., 2006). The theory of Developmental Traumatology (van der Kolk, 2005) outlines how the experience of chronic trauma during childhood and adolescence can affect the physical architecture of the developing brain. It is clear that childhood exposure to abuse can cause chronic trauma, and emerging evidence suggests that there may be certain shorter windows or sensitive periods during which children may be more susceptible to the effects of abuse. Developmental traumatology suggests that chronic trauma may make individuals hypersensitive to stress and lead to a lack of confidence, self-worth, motivation, and aspiration, increased risk of depression and other mental health difficulties, and difficulties in social interaction and relationship formation (De Bellis et al., 2005, 2009; K. Devries et al., 2016). These outcomes are believed to result in a range of functional impairments, including vocational impairment (van der Kolk, 2005). Mechanisms relating to the biological embedding of stress have also been proposed to explain the long-term poor mental health of those experiencing childhood bullying (Danese & McEwen, 2012). Other mechanisms relate to symptoms of poor mental health experienced at the time of bullying, carried into adulthood. However, socioeconomic status, associated with both experience of childhood violence and poor work outcomes, may be an important confounder in relationships between childhood violence and work outcomes (Duncan et al., 2010; Umeda et al., 2015).

Similarly, education is likely to be an important mediator of the association between childhood violence and work outcomes. A systematic review of 67 studies from 21 countries found that early violence experiences are associated with a lower likelihood of graduating from school and poor educational outcomes (Fry et al., 2018). While overall, there were clear associations between experiencing violence in childhood and a range of educational outcomes, associations varied by type of violence experienced; for example, sexual violence resulted in a larger impact on educational attainment, whereas bullying had minimal association with attainment but was strongly associated with absenteeism and school engagement. This variation highlights the different mechanisms through which particular forms of violence may influence outcomes. Evidence is needed to examine the pathways between childhood violence and later work outcomes and determine how interventions may best reduce the negative outcomes of early experiences of violence (Culp et al., 1987; De Bellis et al., 2005).

To address gaps in previous reviews, we conducted a systematic review to:

1. Determine the direction and magnitude of the association between experiences of violence and witnessing violence in childhood and work outcomes in adulthood. Experiences of violence include physical, sexual, and emotional violence, neglect and bullying, and witnessing violence includes witnessing intimate partner violence (IPV) among parents/caregivers, and work outcomes are in the immediate and long term.
2. Identify the evidence gaps in terms of geography, forms of violence, and work outcomes with respect to the association between experiences of violence in childhood and work outcomes.
3. Summarize evidence on the moderation of the relationship between different forms of violence and work outcomes by gender.
4. Summarize evidence on potential mediators of the relationship between different forms of violence and work outcomes.

## Methods

### Inclusion Criteria

Studies were eligible for inclusion if they met the following criteria: (a) a quantitative, prospective longitudinal study with an observational, interventional, or case-control design, and (b) reporting data on experiences of violence in childhood and one or more work outcomes. Studies were considered longitudinal if data on the exposure (violence) were collected prior to data on the work outcome. “Childhood” was defined as between 0 and 18 years. All authors’ definitions of violence and work outcomes were considered. Studies were excluded if using cross-sectional data (or if data on childhood violence and work outcomes were collected at the same time point), synthesized data (i.e., systematic review or other review format), or qualitative data.

### Search Strategy

Figure 1 summarizes the process for searches and screening. Our search terms included words relating to childhood, violence, and work outcomes. We entered these search terms into the following electronic bibliographic databases from first record to 19^th^ August 2019: MEDLINE (Ovid), EMBASE (Ovid), PsycINFO (Ovid), EmCARE (Ovid), Global Health (Ovid), IBSS (Proquest), Web of Science, Scopus and The Cochrane Library (Cochrane Database of Systematic Reviews, Cochrane Methodology Register). Updated searches were conducted on 4^th^ November 2021 and in April and May 2024. No language or date restrictions were used. An example of the search strategy is in Supplemental File 1. We additionally reviewed the reference lists of relevant systematic reviews and meta-analyses.

**Figure 1.**
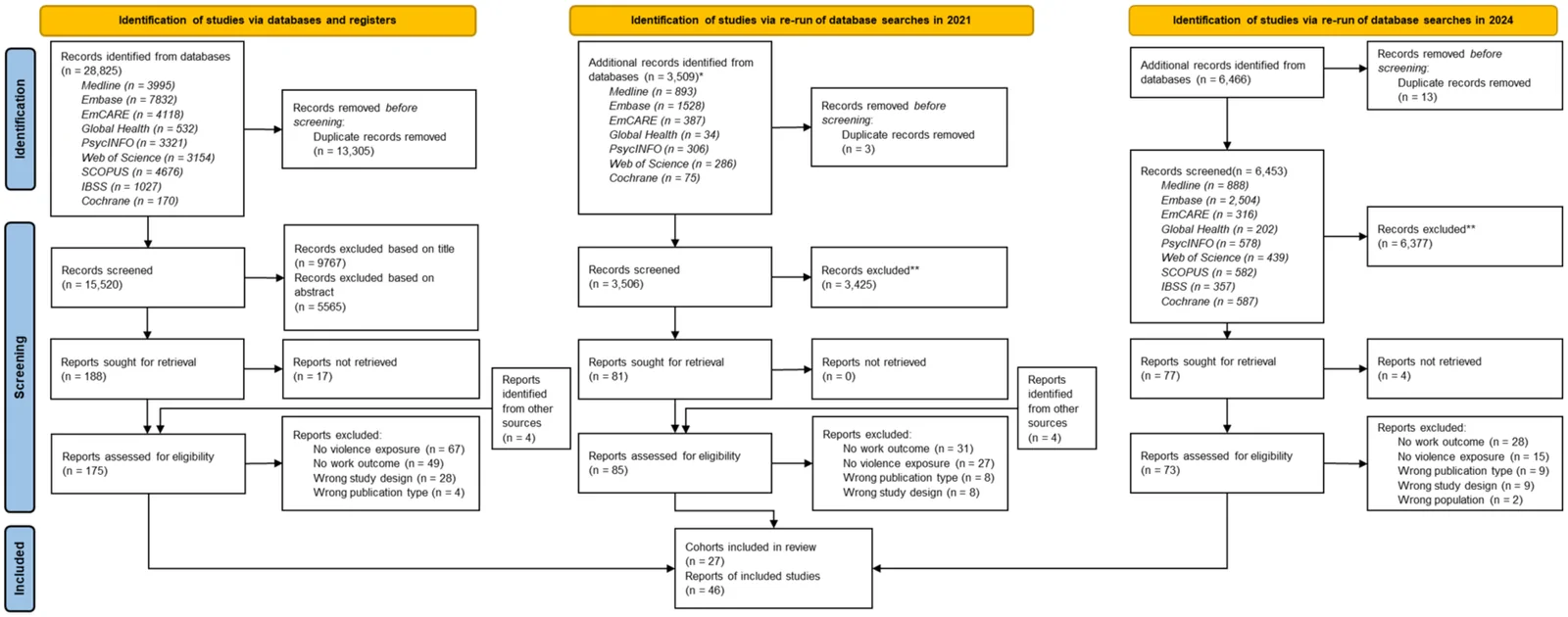
PRISMA 2020 flow diagram for new systematic reviews, which included searches of databases, registers, and other sources. *Source.* Page et al. (2021). For more information, visit: http://www.prisma-statement.org/ *Note*. *IBSS could not be searched in the November 2021 search as LSHTM did not have access to this database, and we could not gain access via another institution due to pandemic access restrictions. Scopus could not be searched in the November 2021 search due to technical reasons.

### Screening, Data Extraction, and Quality Assessment

For the initial search, records were imported into Rayyan (Ouzzani et al., 2016). One reviewer (Lki, KM or CT) performed initial screening to remove irrelevant titles, following double-screening of 50 articles. Six reviewers (KM, CT, AK, FK, LKn, and LKi) performed abstract screening, discussing their application of standardized inclusion criteria on a subset of 20 abstracts. Two reviewers (KM and CT) conducted a quality control screening of 20% of abstracts to ensure consistency. Discrepancies were discussed to reach a consensus. For the updated searches, records were imported into Covidence (Veritas Health Innovation, 2018). Three reviewers performed title/abstract screening (KM, JP, and CT). An initial 10% of records were double-screened, and >97% agreement was achieved. All discrepancies were discussed by the three reviewers to reach a consensus. The remaining titles/abstracts were single-screened.

Three reviewers (KM, JP, and CT) carried out full-text screening, data extraction, and quality assessment. The first 10% of the full texts were double-screened, and reviewers then independently screened the remaining full texts. Uncertainties were discussed by two or more reviewers to reach consensus. For each included study, one reviewer extracted the data into a customized database and another checked the extracted data to confirm accuracy. Any disagreements were resolved after discussion. Data were extracted on both cohort- and study-level characteristics: study setting, design, and population; length of follow-up; exposure and outcome measures; statistical analyses, including whether moderation by gender was assessed; measures of association; mediation (if present); and quality criteria.

Study quality was assessed using a checklist that drew on the JBI Critical Appraisal Checklist for Cohort Studies (Joanna Briggs Institute, 2016), which includes 11 quality measures (e.g., confounding factors identified and appropriate statistical tests used), as well as the Newcastle-Ottawa scale and a scoring system published in Norman et al. to tailor assessment criteria to the review topic. For example, we added a component to capture whether the exposure was assessed during childhood (versus retrospectively recalled in adulthood), since we would expect higher quality measures to be captured closer to the time of the violence experience. The reviewer extracted the data, completed the checklist, and a second reviewer checked the extraction, confirming its accuracy. Scores were attached to each question, so the overall quality score ranged from 0 to 8. We did not formally assess the quality of the mediation analyses and instead focused on describing which mediators have been explored.

### Data Synthesis

We performed a narrative synthesis of findings from the included studies describing the study population, violence exposure, work outcome, and measure(s) of association between exposure and outcome. Data were first grouped according to type of violence: physical violence, sexual violence, emotional violence, neglect, bullying, witnessing violence, substantiated reports of child abuse/neglect, polyvictimization, or composite measures which combined multiple violence types; and then according to work outcome. Polyvictimization was defined by the experience of more than one type of violence (Finkelhor et al., 2007), as measured in the study, while composite measures grouped together more than one type of violence but did not measure whether an individual experienced one or more than one. All relevant exposure-outcome associations are reported in the detailed results table (Supplemental File 2). Binary study outcomes are reported on Forest Plots; where a report gave multiple presentations of the relationship between an exposure and outcome, the most general result was chosen (e.g., not in a particular subgroup). We also described whether estimates were adjusted for confounding by socioeconomic status (SES) or related factors (including parental education) in childhood, but we did not comprehensively consider all confounders, as these would vary by study population and outcome assessed, so it is not possible to determine a universal minimum set. We also described whether studies had adjusted for exposure to other forms of violence, although we did not consider these to be potential confounders because they may be on the causal pathway between initial violence exposure and work outcomes. We provided a narrative synthesis of the relationships examined for moderation by gender and the results of these analyses, and the mediators examined and their results of these analyses. Meta-analyses were not conducted given the heterogeneity of the exposure and outcome measures and differences in the length of follow-up across studies. There were too few studies with the same exposure and the same outcome to make funnel plots to assess publication bias.

The review protocol was published on ResearchGate (https://www.researchgate.net/project/Experience-of-violence-in-childhood-and-work-outcomes-a-systematic-review). This article follows PRISMA reporting standards.

## Results

A total of 38,800 title/abstracts were found through the database searches (Figure 1), with 8 additional reports identified through different methods. In total, 46 reports of 27 cohorts met our inclusion criteria. One report presented results from two cohorts.

### Study Characteristics

Table 1 and Supplemental File 2 provide a summary and detail, respectively, of the cohorts and reports included. Most cohorts were from the USA (*n*=11), with multiple cohorts from the United Kingdom (*n*=4), Canada (*n*=3), and Norway (*n*=2); and one cohort from each of Australia, Denmark, New Zealand, Switzerland, Taiwan, the Netherlands, and South Africa. Six were nationally representative, and 21 recruited a subpopulation. Most cohorts recruited participants before adulthood. Cohort participants were born between the 1940s and 1990s. Length of follow-up ranged from 15 months to 55 years.

**Table 1.** Descriptive Overview of Included Cohorts and Reports.

Characteristic	***N***	**%**
By cohort (*n*=27)		
Country	27	100.0%
USA	11	40.7%
UK	4	14.8%
Canada	3	11.1%
Norway	2	7.4%
Australia	1	3.7%
Denmark	1	3.7%
New Zealand	1	3.7%
Switzerland	1	3.7%
Taiwan	1	3.7%
Netherlands	1	3.7%
South Africa	1	3.7%
Population	27	100.0%
Nationally representative	6	22.2%
Subpopulation	21	77.8%
Age at enrollment	28	100.0%
Birth	6	21.4%
0–11 years	9	32.1%
9, 11, 13 years	1	3.6%
11–18 years	8	28.6%
≥ 18 years	2	7.1%
12–21 years	1	3.6%
14–22 years	1	3.6%
Year when participants born*	38	100.0%
1940s	1	2.6%
1950s	5	13.2%
1960s	4	10.5%
1970s	7	18.4%
1980s	12	31.6%
1990s	8	21.1%
Before 1973	1	2.6%
**By report (*n*=46)**		
Violence exposures	73	100%
Composite measures	14	19.2%
Bullying	12	16.4%
Physical violence	10	13.7%
Sexual violence	9	12.3%
Child abuse and neglect (official records)	8	11.0%
Neglect	6	8.2%
Emotional violence	4	5.5%
Witnessing violence	4	5.5%
Polyvictimization	3	4.1%
IPV	3	4.1%
Reporting of exposure	65	100.0%
Self-report	43	66.20%
Administrative records	9	13.80%
Parent-report only	6	9.20%
Self-, parent-, and teacher report	2	3.10%
Teacher-report only	1	1.50%
Self-report and parent-report	3	4.60%
Medical-examiner report	1	1.50%
Data collection for exposure	62	100.0%
Prospective (during childhood)	44	71.0%
Retrospective (during adulthood)	13	21.0%
Mix of prospective and retrospective	5	8.1%
Work outcome	53	100.0%
Income	23	37.7%
Employment status	22	36.10%
Benefits	10	16.4%
Occupational prestige	6	9.8%
NEET	8	13.1%
Occupational classification	4	6.6%
Work trajectory	4	6.6%
Combined measure	2	3.3%
Work ability	2	3.3%
Motivation	2	3.3%
Sickness absence	1	1.6%
Assessed moderation by sex	47	100.0%
Yes	12	25.5%
No	35	74.5%
Disaggregated data by sex	47	100.0%
Yes	11	23.4%
No	36	76.6%
Assessed mediation	47	100.0%
Yes	9	19.1%
No	38	80.9%
Mediators assessed	16	100.0%
Education	5	31.3%
Combination	3	18.8%
Health	3	18.8%
Cognition	2	12.5%
Family-related factors	1	6.3%
Work-related factors	1	6.3%
IPV	1	6.3%
Other	1	6.3%
Quality (score out of 8)	47	100.0%
4	4	8.5%
4.5	3	6.4%
5	9	19.1%
5.5	4	8.5%
6	10	21.3%
6.5	7	14.9%
7	8	17.0%
7.5	0	0.0%
8	2	4.3%

In the section by report, reports are included more than once if the report falls into multiple categories.

Macmillan (1998) includes two cohorts and is therefore included twice in the sections by report.

* Cohorts included more than once if the cohort includes individuals born in multiple decades

Occupational classification includes manual occupations, skilled, and selling sexual services. Work trajectory includes income trajectory, occupational prestige trajectory, and intra- and inter-generational social mobility. Combined includes economic hardship, individual-level SES (based on income, public assistance, and unemployment). Work ability includes dismissal from a job, poor interview-rated social and occupational functioning. Motivation includes quitting multiple jobs and lack of long-range career goals.

Reports examined a total of 73 violence exposures. Composite measures (e.g., child maltreatment, victimization) were most commonly examined (*n*=14), followed by bullying (*n*=12), physical violence (*n*=10), and sexual violence (*n*=9). Other exposures included child abuse and neglect (CAN) from official records (*n*=8), neglect (*n*=6), emotional violence (*n*=4), witnessing violence (*n*=4), IPV (*n*=3), and polyvictimization (*n*=3). Exposures were mostly self-reported (*n*=43) rather than from official records (*n*=9). Other exposures were reported by parents, teachers, or combinations. Most exposures were measured prospectively during childhood or late adolescence up to age 18 years (*n*=44), with 13 collected retrospectively when participants were over 18 years, and five using both prospective and retrospective components. Data on work measures were collected from participants aged 17 to 55 years old across studies. A total of 61 work outcomes were examined, most commonly income (*n*=23) and employment status (*n*=22) or related outcomes including benefits, occupational classification (e.g., sex work), not in employment, education or training (NEET) (*n*=28), but also work trajectories (*n*=3), work ability (*n*=2), motivation (*n*=2), sickness absence (*n*=1) and a combined measure (*n*=2). No reports examined child labor/trafficking as a work outcome. Around one-third of reports examined moderation by gender, and one-third examined mediation. Mediator variables were assessed at varying timepoints, including contemporaneously with exposure or outcome measurements in some cases.

Reports were medium to high quality, with scores ranging from 4 to 8 on the 0 (low quality) to 8 (high quality) scale. Generally, reports gained points for using representative populations, conducting appropriate statistical analyses, and ensuring appropriate reporting of violence. Reports generally scored lower for having follow-up below 80%, not adjusting for childhood SES, and not asking multiple questions about violence acts.

### Association Between Violence and Work Outcomes

Results for binary outcomes are presented in Figure 2 and continuous outcomes in Figure 3. Detailed results from each report are presented in Supplemental File 3. Results are presented by violence exposure. Looking across all reports, there is good evidence that exposure to violence in childhood is associated with subsequent work outcomes. Associations are found across multiple types of violence, although evidence is strongest for official reports of CAN, physical violence, bullying, and composite measures of violence. There is little evidence for adolescent IPV. Associations exist in relation to a range of work outcomes, and evidence is strongest for income and employment status. There is less evidence for a relationship with occupational classification.

**Figure 2.**
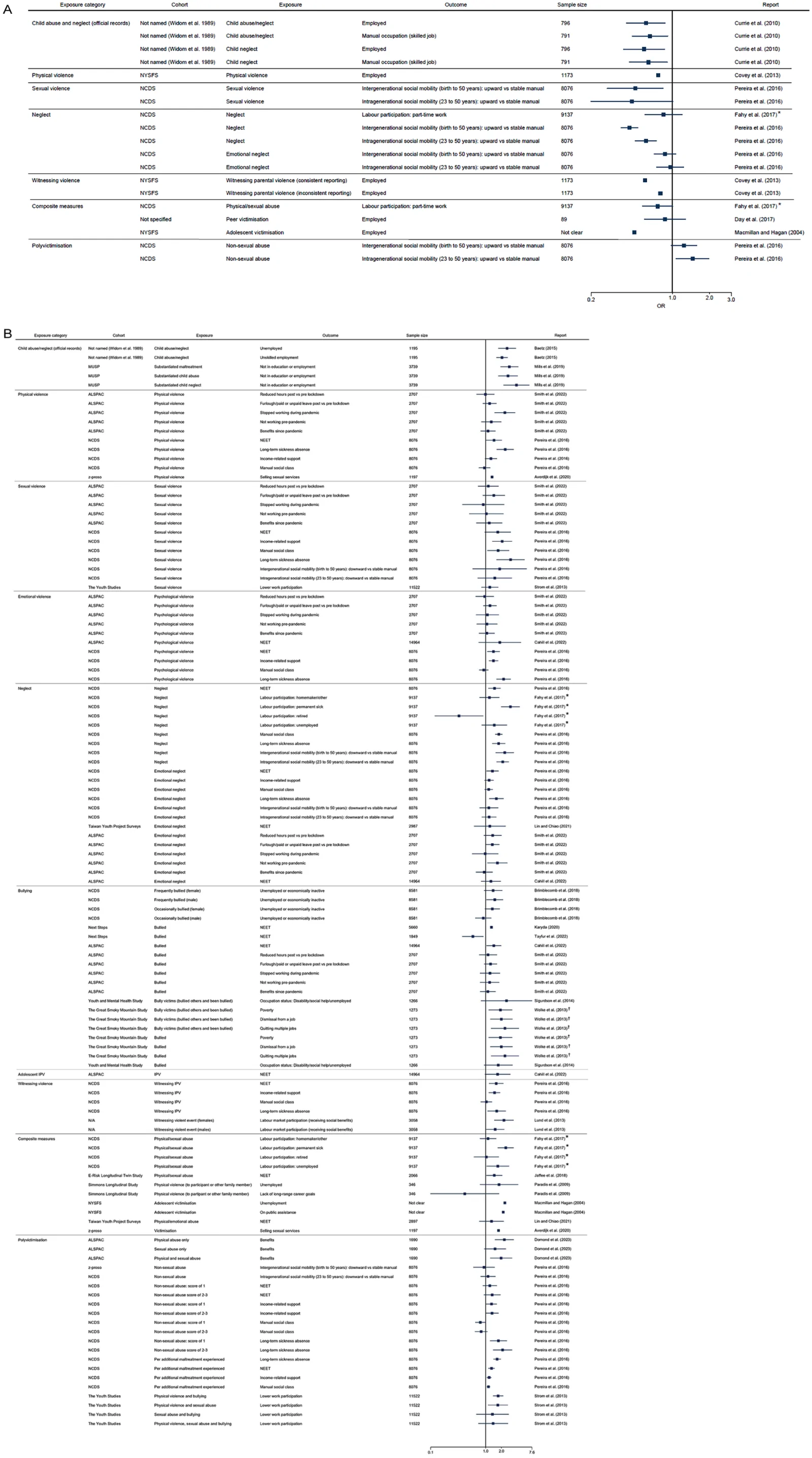
Association between violence and binary work outcomes. 2A: Association between violence and binary positive work outcomes. 2B: Association between violence and binary negative work outcomes. *Note*. ALSPAC=Avon Longitudinal Study of Parents and Children; MUSP=Mater-University of Queensland Study of Pregnancy; NCDS=National Child Development Study; NYSFS=National Youth Survey Family Study; NEET=not in employment, education or training; z-proso=Zurich Project on the Social Development from Childhood into Adulthood *Fahy et al. (2017) measure association using the risk ratio. All other studies measure association using odds ratio. †Crude ORs are presented; no adjusted OR is presented in the original paper There were no reported associations for the following exposure-outcome relationships and therefore these have been excluded from the forest plot (Figure 2A: Gibb et al., 2012: physical abuse-welfare dependence, physical abuse-economic hardship, sexual abuse-welfare dependence, sexual abuse-economic hardship. Figure 2B, Gibb et al., 2012: physical abuse-employment, sexual abuse-employment; Tam et al., 2003: physical abuse-labor force participation, sexual abuse-labor force participation) We presented the most adjusted analysis from the papers, except for when there were additional adjustments, which included something that may be on the causal pathway, when we then used the most adjusted analysis without this.

**Figure 3.**
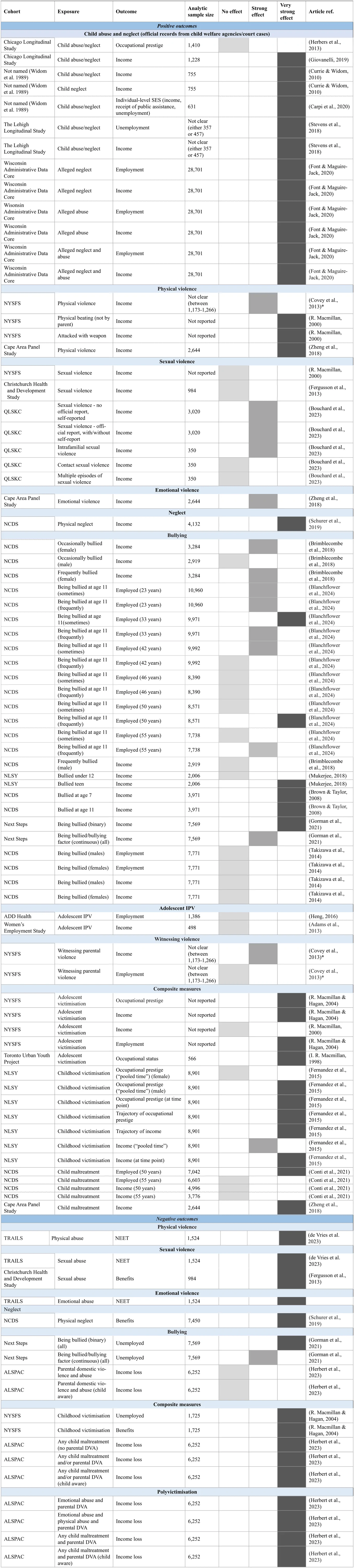
Association between measures of violence against children and continuous work outcomes. Abbreviations: ADD Health, National Longitudinal Study of Adolescent to Adult Health; ALSPAC, Avon Longitudinal Study of Parents and Children; MUSP, Mater-University of Queensland Study of Pregnancy; NCDS, National Child Development Study; NLSY, National Longitudinal Survey of Youth 1997; NYSFS, National Youth Survey Family Study; QLSKC, Quebec Longitudinal Study of Kindergarten Children; SES, socioeconomic status; z-proso, Zurich Project on the Social Development from Childhood into Adulthood; TRAILS, TRacking Adolescents’ Individual Lives Survey Measures of association are detailed in Supplemental File 3 This figure does not include results from the National Youth Survey Family Study (NYSFS) from Macmillan et al. (1998), as similar analyses with the same dataset are reported in Macmillan and Hagan (2004). Results of Macmillan et al. (1998) are detailed in Supplemental File 3 Strong effect is defined as *p*<0.1, very strong effect defined as *p*<0.01. There are two analyses where no measure of association is presented (Gibb et al., 2012, for exposures: physical abuse by parent, sexual abuse; for outcome; income) *Only consistent reporting of violence is included in the figure. Both consistent and inconsistent reporting are included in Supplemental File 3.

#### Child Abuse and Neglect (Official Records From Child Welfare Agencies/Court Cases)

In eight reports from five cohorts, official records were used to define the exposure. In all reports, those who had experienced CAN were more likely to have negative work outcomes (Baetz, 2015; Carpi et al., 2020; Currie & Widom, 2010; Font & Maguire-Jack, 2020; Giovanelli, 2019; Herbers et al., 2013; Mills et al., 2019; Stevens et al., 2018), and in reports examining neglect and abuse separately, similar associations with negative work outcomes were seen (Currie & Widom, 2010; Font & Maguire-Jack, 2020; Mills et al., 2019). In four reports, associations were adjusted for childhood SES or related factors (Currie & Widom, 2010; Font & Maguire-Jack, 2020; Giovanelli, 2019; Herbers et al., 2013).

#### Physical Violence

Nine reports from eight cohorts examined the association between physical violence and work outcomes. There is evidence from these reports for a negative relationship between physical violence and income (Covey et al., 2013; Zheng et al., 2018), and positive relationships with unemployment (Tam et al., 2003; Zheng et al., 2018), income deficit/support (R. Macmillan, 2000), long-term sickness absence (Pinto Pereira et al., 2017), NEET status (De Vries et al., 2023; Pinto Pereira et al., 2017) and stopping working during the COVID-19 pandemic (Smith et al., 2022). There is no evidence for a relationship with manual occupation or lack of long-range career goals, examined in only one study, or other changes in employment post-COVID-19 lockdown compared to pre-lockdown in the United Kingdom, including reduced hours, stopping working or claiming benefits (Smith et al., 2022). One report did not present an association but found physical violence to be significant in the regression model for the effect of childhood family income on working in paid employment, but not income, welfare dependence of economic hardship (Gibb et al., 2012). Four reports adjusted for childhood SES or similar markers (Covey et al., 2013; R. Macmillan, 2000; Pinto Pereira et al., 2017; Zheng et al., 2018).

#### Sexual Violence

Eight reports from seven cohorts reported on the association between sexual violence and work outcomes. There was evidence that experience of sexual violence in childhood is negatively associated with occupational classification (Bouchard et al., 2023) and positively associated with income support (Pinto Pereira et al., 2017), long-term sickness absence (Pinto Pereira et al., 2017), and being NEET (De Vries et al., 2023; Pinto Pereira et al., 2017). Those who had experienced sexual violence were also less likely to have an upward intra- and inter-generational social mobility (Pinto Pereira et al., 2017). The evidence was mixed for an association between sexual violence and income (Bouchard et al., 2023; Fergusson et al., 2013). One report did not present an association but found sexual violence to be significant in the regression model for the effect of childhood family income on welfare dependence and economic hardship, but not income or paid employment (Gibb et al., 2012). There was no evidence for an association between sexual violence and changes in employment or benefit receipt since the COVID-19 pandemic (Smith et al., 2022). Four reports adjusted for childhood SES or similar markers (Bouchard et al., 2023; Fergusson et al., 2013; R. Macmillan, 2000; Pinto Pereira et al., 2017; Smith et al., 2022). Two reports adjusted for other types of violence (Fergusson et al., 2013; Pinto Pereira et al., 2017), of which one adjusted only when looking at the association with social mobility outcomes (Pinto Pereira et al., 2017).

#### Emotional Violence

Evidence on emotional violence came from five reports of four cohorts. In one, those who had experienced emotional violence had lower income, but there was no association with employment (Zheng et al., 2018). There was no evidence of an association with changes in work outcomes since the COVID-19 pandemic (Smith et al., 2022). The other reports suggested that those who had experienced emotional violence were more likely to be on long-term sickness absence, NEET, and on income-related support, but there was no relationship with manual occupation (Cahill et al., 2022; De Vries et al., 2023; Pinto Pereira et al., 2017). Four reports adjusted for childhood SES or similar markers (Cahill et al., 2022; Pinto Pereira et al., 2017; Smith et al., 2022; Zheng et al., 2018).

#### Neglect

Five reports from three cohorts reported on the association between neglect and various work outcomes. Reports examined neglect (Fahy et al., 2017; Pinto Pereira et al., 2017), physical neglect (Schurer et al., 2019), and emotional neglect (Cahill et al., 2022; Lin & Chiao, 2022; Pinto Pereira et al., 2017; Smith et al., 2022). Those who had experienced neglect were more likely than those who had not experienced neglect to be NEET, experience long-term and permanent sickness absence, be in a manual occupation, and experience downward inter- and intra-generational social mobility, though no association was seen with income-related support (Pinto Pereira et al., 2017). Associations tended to be weaker for emotional neglect (Cahill et al., 2022; Lin & Chiao, 2022; Smith et al., 2022). Four reports adjusted for markers of childhood SES (Cahill et al., 2022; Pinto Pereira et al., 2017; Schurer et al., 2019; Smith et al., 2022) and one for education markers, although variables on the causal pathway may have inadvertently been adjusted for (e.g., educational aspiration and class rank) (Lin & Chiao, 2022). One report adjusted for other types of violence when looking at social mobility outcomes (Pinto Pereira et al., 2017).

#### Bullying

Data on the relationship between bullying and work outcomes comes from twelve reports from six cohorts. There is evidence that those bullied were more likely to experience negative work outcomes including unemployment, NEET, poverty, job dismissal, quitting multiple jobs, and lower income (Blanchflower & Bryson, 2024; Brimblecombe et al., 2018; Brown & Taylor, 2008; Cahill et al., 2022; Gorman et al., 2021; Mukerjee, 2018; Wolke et al., 2013). Where reports also looked at bully-victims, associations were generally similar (Sigurdson et al., 2014; Wolke et al., 2013). Findings from one study that looked at employment at different ages suggested stronger associations with employment outcomes at 23, 33, and 42 years compared to 46, 50, and 55 years. There was no evidence of an association between being bullied and changes in employment post-COVID-19 (Smith et al., 2022). Ten reports adjusted for childhood SES or related factors (Brimblecombe et al., 2018; Brown & Taylor, 2008; Cahill et al., 2022; Gorman et al., 2021; Karyda, 2020; Mukerjee, 2018; Sigurdson et al., 2014; Smith et al., 2022; Takizawa et al., 2014; Tayfur et al., 2022). Two reports adjusted for other types of violence included within their definition of adversity (Brimblecombe et al., 2018; Takizawa et al., 2014).

#### Adolescent IPV

Three reports from three cohorts reported on the association between adolescent IPV and employment status, NEET, and earnings. Earnings were lower in those who had experienced adolescent IPV (Adams et al., 2013), but there was weaker evidence for a relationship with NEET (Cahill et al., 2022), and no relationship with employment status (Heng, 2016). Only one adjusted for childhood SES (Cahill et al., 2022).

#### Witnessing Violence

Four reports from four cohorts examined the association between witnessing violence in childhood and various work-related outcomes. In three reports, there was evidence that witnessing violence increased the likelihood of negative work outcomes (Covey et al., 2013; Lund et al., 2013; Pinto Pereira et al., 2017), although this evidence was weaker in one study (Covey et al., 2013). One study suggested no association between parental domestic violence and loss of earnings, regardless of whether the child was aware of the violence or not (Herbert et al., 2023). Three reports adjusted for measures of childhood SES (Covey et al., 2013; Lund et al., 2013; Pinto Pereira et al., 2017)

#### Composite Measures

There were fourteen reports from twelve cohorts that used composite violence measures. Violence measures included childhood victimization (Fernandez et al., 2015; I. R. Macmillan, 1998), adolescent victimization (R. Macmillan, 2000; R. Macmillan & Hagan, 2004), violent victimization (Averdijk et al., 2020), peer victimization (Day et al., 2017), child abuse (Lund et al., 2013), child maltreatment (Conti et al., 2021), family physical violence (Paradis et al., 2009), physical/sexual abuse (Fahy et al., 2017; Jaffee et al., 2018), physical/emotional abuse (Lin & Chiao, 2022; Zheng et al., 2018) and child maltreatment and/or parental domestic violence (Herbert et al., 2023). These reports provided evidence for negative associations with income (Fernandez et al., 2015; R. Macmillan & Hagan, 2004; Zheng et al., 2018), employment (Conti et al., 2021; Covey et al., 2013; R. Macmillan & Hagan, 2004), and trajectories of occupational prestige and income (Fernandez et al., 2015); and positive associations with unemployment (I. R. Macmillan, 1998; R. Macmillan & Hagan, 2004), income support (I. R. Macmillan, 1998), NEET status (Jaffee et al., 2018), selling sexual services (Averdijk et al., 2020) and loss of earnings (Herbert et al., 2023). Eight adjusted for childhood SES or related markers (Conti et al., 2021; Jaffee et al., 2018; Lund et al., 2013; I. R. Macmillan, 1998; R. Macmillan, 2000; R. Macmillan & Hagan, 2004; Paradis et al., 2009; Zheng et al., 2018). One study adjusted for other types of violence, specifically IPV experienced as a young person (Herbert et al., 2023).

#### Polyvictimization

Four reports from four cohorts presented findings on polyvictimization. In one (Strøm et al., 2013), which asked about physical violence, sexual abuse, and bullying, after adjustment for SES markers, associations with not participating in work were stronger for those experiencing more forms of violence, but estimates were imprecise. The other study created two polyvictimization measures (Pinto Pereira et al., 2017), one combining different forms of violence (physical abuse, psychological abuse, and witnessing IPV) and creating a score for the number experienced, and the other looking at number of maltreatment types, also including other maltreatment (e.g., neglect). After adjusting for childhood SES markers, there was evidence for a stronger association for each additional maltreatment type experienced for all negative work outcomes (long-term sickness, NEET, income-related support, and manual occupation). There was less evidence for stronger associations with multiple forms of violence, with larger ORs only for long-term sickness and NEET. The authors also adjusted for other types of violence when looking at the association between multiple forms of violence and social mobility outcomes (Pinto Pereira et al., 2017). Another study measured the association between experiencing both physical and sexual abuse, as well as physical or sexual only, and receiving welfare benefits. After adjusting for makers related to SES, they found similarly strong evidence for an association between those reporting physical abuse only to those reporting both physical and sexual abuse (Domond et al., 2023). The fourth report found strong evidence of a relationship between experiencing emotional abuse, emotional and physical abuse, or any child maltreatment, alongside parental domestic violence, and increased loss of earnings (Herbert et al., 2023). This was not adjusted for SES, but was adjusted for being subject to IPV as a young person.

#### Severity of Exposure

In six reports, violence exposures were analyzed using categorical variables to denote severity. One reported on two types of physical and emotional violence (Zheng et al., 2018), two on the nature of sexual violence (Bouchard et al., 2023; Fergusson et al., 2013), and four on bullying (Blanchflower & Bryson, 2024; Brimblecombe et al., 2018; Brown & Taylor, 2008; Gorman et al., 2021). Where associations existed, stronger relationships were generally observed for more frequent/severe violence, though one study found no evidence for a difference between earnings following experience of multiple versus a single episode of, or contact versus noncontact, sexual abuse (Bouchard et al., 2023)

### Moderation by Gender

Of the 37 reports, twelve examined moderation by gender (Averdijk et al., 2020; Covey et al., 2013; Fahy et al., 2017; Fergusson et al., 2013; Fernandez et al., 2015; Gorman et al., 2021; Heng, 2016; Jaffee et al., 2018; Lund et al., 2013; I. R. Macmillan, 1998; Paradis et al., 2009; Pinto Pereira et al., 2017) across a range of exposure-outcome relationships. Only two of these twelve found evidence for moderation in one or more relationships (Averdijk et al., 2020; Fernandez et al., 2015). Gender was found to modify the association between childhood victimization and occupational prestige, where an association was found for males but not females (Fernandez et al., 2015). However, there was no moderation for other analyses in this paper. Gender was also found to moderate the association between violence victimization and selling sexual services (Averdijk et al., 2020), where at higher levels of victimization, females had a higher likelihood of selling sexual services.

An additional seven reports presented results disaggregated by gender, although in most cases this was *a priori* rather than as a result of formal assessment of effect moderation (Baetz, 2015; Bouchard et al., 2023; Brimblecombe et al., 2018; Currie & Widom, 2010; Lin & Chiao, 2022; Mukerjee, 2018; Takizawa et al., 2014; Zheng et al., 2018). From these, there was no evidence for gender modification in three reports of bullying (Brimblecombe et al., 2018; Lund et al., 2013; Mukerjee, 2018), in one report of official records of CAN (Baetz, 2015), or in one report of severity and chronicity of sexual abuse (Bouchard et al., 2023) In three reports associations were stronger or only present for girls with exposures of CAN (Currie & Widom, 2010), physical/emotional abuse (Lin & Chiao, 2022), physical and emotional violence (Zheng et al., 2018) and intrafamilial versus extrafamilial abuse (Bouchard et al., 2023). None of these reports carried out formal interaction tests.

### Mediation

Nine reports (19%) conducted mediation analyses for the exposure-outcome relationship we were interested in (Adams et al., 2013; Brimblecombe et al., 2018; Fernandez et al., 2015; Heng, 2016; Karyda, 2020; I. R. Macmillan, 1998; R. Macmillan, 2000; Pinto Pereira et al., 2017; Schurer et al., 2019; Strøm et al., 2013). In total, there were 57 mediation analyses assessing 56 variables as mediators. Almost 40% of mediators assessed related to education (*n*=14), including educational attainment, performance, investment, and effort. Other variables assessed as mediators related to physical and/or mental health (*n*=10), cognition (*n*=9), family factors (*n*=4), work factors (*n*=2), IPV (*n*=2), combinations of these factors (*n*=13), and others (*n*=2), which included noncognitive skills. Figure 4 summarizes studies showing evidence for mediation of the relationship between violence against children and work outcomes. Supplemental File 4 details mediation results from all studies assessing mediation. We summarize the results according to the main mediating variables examined below.

**Figure 4.**
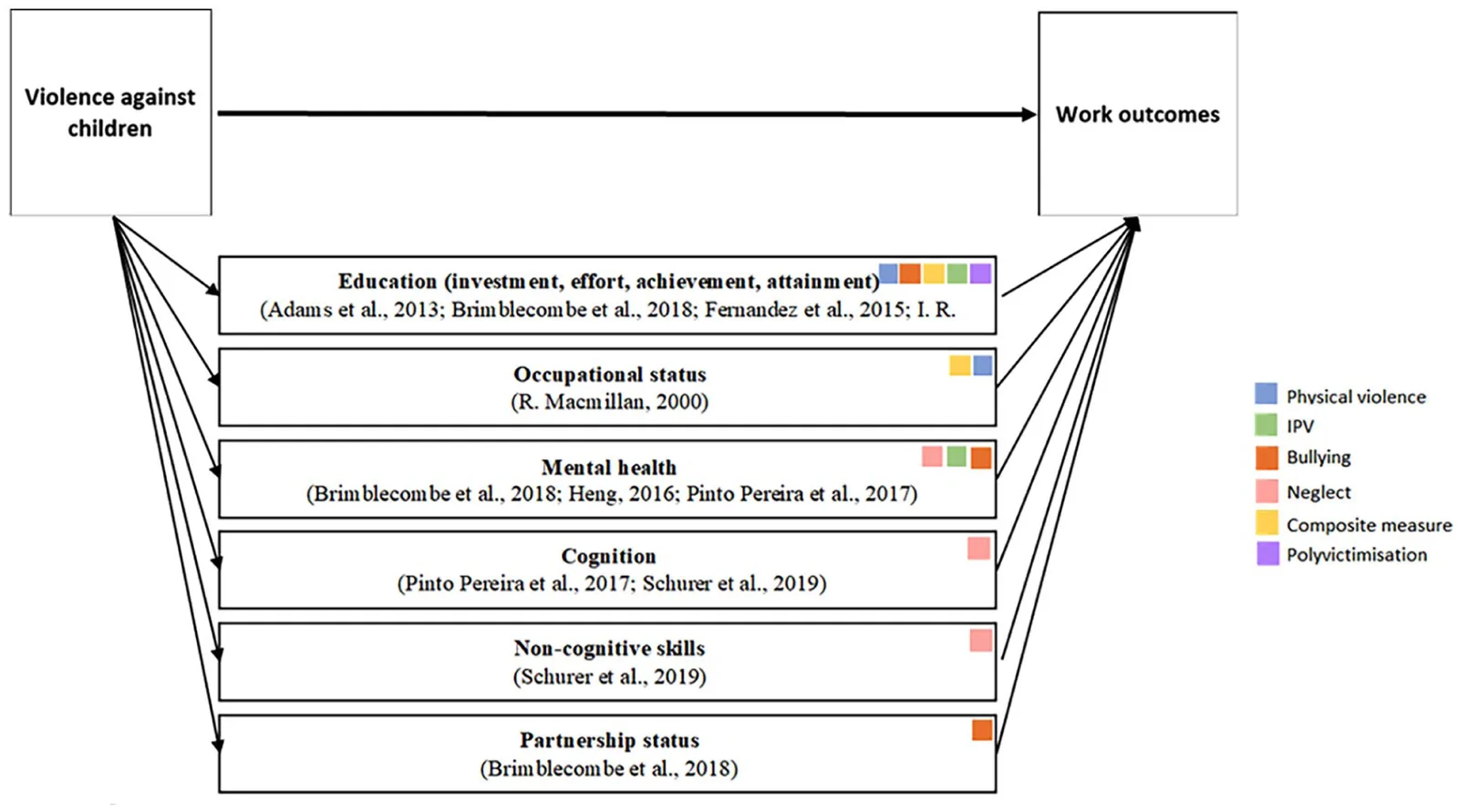
Evidence for mediation of the relationship between violence against children and work outcomes. Analyses included in figure if there was evidence for mediation. Additional mediation analyses were conducted in one report (macmillan 1998) for the relationship between adolescent victimisation and average hourly wage, but results were not legible in the available documentation. Full mediation results can be found in Supplementary Table 3.

#### Education

Of 14 analyses (5 reports) examining education mediators, 7 found education to be a mediator of the relationship between childhood violence and work outcomes. A range of exposures and outcomes were examined, and education was found to be a mediator for income (Adams et al., 2013; Fernandez et al., 2015; Schurer et al., 2019), benefits (Schurer et al., 2019; Strøm et al., 2013), employment status (I. R. Macmillan, 1998), and occupational prestige (Fernandez et al., 2015). This was for a range of violence exposures.

#### Physical and/or Mental Health

Of ten analyses (three reports) examining health as a mediator (Heng, 2016; Pinto Pereira et al., 2017; Schurer et al., 2019), none examining physical health found evidence of mediation. However, one of the ten analyses on mental health found evidence for mediation. Depression mediated the relationship between IPV and employment status (Heng, 2016).

#### Cognition

Analyses of cognition (two reports), which both measured cognitive skills using mathematics and reading test scores, found evidence of mediation. Both reports looked at neglect as the exposure, with one study looking at earnings and welfare dependence as the outcome (Schurer et al., 2019) and the other at long-term sickness absence (Pinto Pereira et al., 2017).

#### Family-Related Factors

One of four analyses (1 report) examining family-related factors found evidence for partial mediation. The report found that partnership status partially mediated the relationship between physical neglect and welfare dependence, but not earnings. Other family-related factors (including marital status and number of children) were not mediators of the relationship between physical neglect and either earnings or welfare dependence (Schurer et al., 2019).

#### Work-Related Factors

One of two analyses (1 report) examining a work-related mediator found evidence of mediation (Schurer et al., 2019). The report found that labor force attachment partially mediated the relationship between physical neglect and welfare dependence (Schurer et al., 2019).

#### IPV

Neither of the two analyses (1 report) found evidence for mediation by IPV (Heng, 2016).

#### Other/Combined

Both analyses (one report) of noncognitive skills, measured by self-efficacy, found that they mediated the relationship between physical neglect and welfare dependence, and to a weak extent, earnings (Schurer et al., 2019). Seven analyses (three reports) tested the effects of a combined model of mental health and cognition, which was found to mediate the relationship between neglect and both long-term sickness absence and NEET (Pinto Pereira et al., 2017). Other combinations of mediators were found to mediate specific exposure-outcome relationships (Brimblecombe et al., 2018; R. Macmillan, 2000).

## Discussion

### Summary of Key Findings

We identified a body of evidence on the association between several different violence exposures and a range of work outcomes, including outcomes beyond employment status, such as income, receiving benefits, job classification, and social mobility, as well as job dismissal, job aspiration, and sickness absence. With the exception of one South African study, this body of evidence is solely from high-income countries. We found good evidence that official reports of CAN, physical violence, and bullying, as well as composite measures of violence are associated with a range of negative work outcomes, with strong associations seen particularly for reports using exposures derived from official records. Evidence is less clear for sexual violence and is lacking for emotional violence, witnessing violence and neglect, although available evidence also suggests they are associated with negative work outcomes. There are few reports and little evidence to support associations between adolescent IPV and work outcomes. Most reports adjusted for markers of childhood SES, which may be strongly associated with work outcomes. Reports synthesized do not generally provide evidence for different results for men and women, but where results differed by gender, there tended to be stronger associations between violence and adverse work outcomes for women. Although only 23% of reports conducted mediation analyses, there is evidence that some of the association between violence and adverse work outcomes is mediated through education, mental health, cognition, and noncognitive skills. This is strongest for education, for which most research has been done, but further research is needed to explore potential mediators. Table 2 summarizes key findings of our review.

**Table 2.** Critical Findings.

Critical Findings
We identified 46 reports of 27 cohorts that reported on the association between violence in childhood and work outcomes, with all but one from high-income countries.
There is strong evidence from high-income countries that experiencing most forms of violence in childhood is associated with a range of negative work outcomes, including lower income and unemployment.
Evidence does not generally suggest that the association between childhood violence and work outcomes differs between men and women.
Evidence suggests that educational factors may partially mediate the relationship between violence and negative work outcomes. Cognition, mental health, and noncognitive skills may also be mediators.

### Strengths and Limitations

Our review used a comprehensive set of search terms for both violence victimization and work outcomes and searched a range of databases. Although we used a wide range of terms around work, we did not conduct a comprehensive search for literature related to criminality, which may, nevertheless, be considered a work pathway, and therefore did not include reports examining this outcome; however, existing reports suggest a strong association between childhood trauma histories and prison (Teplin et al., 2002). Our full-text screening was only conducted by a single reviewer, so it is possible that we missed some eligible articles. However, screeners met regularly during full-text screening to discuss any uncertainty, so this is unlikely. Initial quality control of the abstract screening revealed some inconsistency, with too many papers included for full-text screening. Extra quality control was conducted to ensure confidence in the process. We scored reports generally as being of high quality. However, some reports did not adjust for confounders, and moderation was reported in less detail. Only 23% of reports conducted mediation analyses. We also acknowledge the inherent difficulties in effectively measuring and modeling violence exposure, which can be chronic, episodic, come in multiple forms, and may be under-reported, especially if stigmatized. In all studies, it is likely that some misclassification of violence exposure, which may bias estimates either toward or away from the null.

### Comparison With Other Literature

Our review supports and extends other studies that point to a long-term impact of most forms of violence in childhood on education as well as socioeconomic outcomes. Our review adds to these previous reviews (Bunting et al., 2018; Fry et al., 2018) by providing evidence for a relationship with other work outcomes—including dismissal from a job, quitting multiple jobs, occupational prestige, and sickness absence—and documents the current evidence around potential mediators for these relationships. Although overall we find strong evidence that experience of violence in childhood is associated with a range of work outcomes, associations do differ depending on the exact relationship examined. For example, we find evidence for a relationship between sexual violence and income support, but not with employment/unemployment or income. Reviews of socioeconomic and educational outcomes also find differences by violence type (Bunting et al., 2018; Fry et al., 2018).

Our findings also highlight the importance of polyvictimization and the severity of violence as a risk factor for negative work outcomes. Other studies have shown that this accumulation of exposures may have a greater negative impact on individuals than single experiences or less severe forms of exposure (Finkelhor et al., 2007; Holt et al., 2007; Ramos de Oliveira & Jeong, 2021). We found particularly strong associations between official records of CAN and work outcomes. Violence defined according to official records, may represent experiences of chronic trauma. The theory of developmental traumatology suggests an impact on neurobiological development leading to developmental delays in cognition, language, motor, and socialization skills. Although there were no mediation analyses for the relationship between official records of CAN and work outcomes, other analyses find evidence for mediation through educational attainment, mental health, and cognition, which is in line with this theory. Education is known to be a major predictor of income, unemployment, and receiving welfare (Loprest & Zedlewski, 1999; Na & Cheng, 2007; Zhan & Pandey, 2004).

### Implications for Research and Policy

Table 3 summarizes the implications of the review for practice, policy, and research. There is a need for longitudinal data from LMICs. The prevalence of childhood violence is higher in many LMICs relative to HIC countries, and social norms around the acceptability of violence may also differ. The cultural normativeness hypothesis states that the psychosocial impact of violence may be less where violence is more culturally normative (Lansford et al., 2005). Under this theory, it is possible that childhood violence exposures are less likely to result in negative work outcomes in settings where violence is more prevalent. Conversely, literature on the biology of trauma suggests that the negative impacts of violence are embodied and lead to poor outcomes regardless of how violent events are perceived (van der Kolk, 2005).

**Table 3.** Implications of the Review for Practice, Policy, and Research.

Review Implications
This review re-emphasizes the need for interventions to prevent violence in childhood. Parenting interventions in the home, as well as school-based interventions to reduce bullying and sexual violence, may have long-reaching consequences on an individual's work outcomes.
Secondary prevention efforts to support children to achieve a good standard of education are important in reducing the long-term effects of experiencing childhood violence on later work outcomes.
Work environments themselves may provide opportunities for secondary prevention.
There is a need for longitudinal data from low- and middle-income countries to understand whether the same relationship between violence in childhood and negative work outcomes exists in contexts where the prevalence of violence and nature of the labor market differ, in addition to the factors that may explain the association between violence and work outcomes.
Further mediation analyses that are context-specific are needed to explore other individual-level factors that might be amenable to interventions that aim to mitigate the consequences of violence.

Access to and the nature of the labor market in LMICs are also different, and there are other negative work outcomes like child labor and hazardous work, which are more common in these settings and for which longitudinal data is scarce. However, a recent analysis of cross-sectional data from the national Violence Against Children surveys in nine LMICs found associations in around half of the countries between violence in childhood and working in a hazardous sector among those aged 18 to 24 years (Bhatia et al., 2022).

Evidence suggests that the effects of violence may be partially mediated through impacts on education. Secondary prevention efforts that support children to achieve a good standard of education are important to reduce the long-term effects of experiences of violence in childhood on individuals’ work and socioeconomic outcomes. Schools can play an important role in this; for example, evidence suggests school attachment and motivation to succeed may mediate the relationship between violence and academic achievement (Sonsteng-Person et al., 2023). In low-resource settings, it may also be that different mediating factors are important for secondary prevention of adverse sequelae. For example, in settings where education systems are under-resourced and children experience high levels of violence in school, education may not be a strong mediator of the association between violence and work outcomes. More context-specific research is needed to identify mediators that may be appropriate targets for secondary prevention. Further mediation analyses are needed to explore the role of interpersonal skills and other individual-level factors that might be amenable to interventions attempting to mitigate the consequences of violence. Research suggests that children have the capacity to cope with difficult circumstances and successfully recover from trauma (Panter-Brick, 2003; Ungar, 2011). However, this capacity can be greatly influenced by the socio-ecological factors that may shape the various health and development realities (Coren et al., 2013; Masten, 2001). Interventions around some of the mediators identified through this review, for example, mental health and cognitive skills (Lindert et al., 2020; Lorenc et al., 2020), as well as trauma-informed teaching techniques, may help reduce some of the negative impacts of violence (Fondren et al., 2020; Gerke et al., 2019; Zakszeski et al., 2017).

Work environments themselves may provide opportunities for secondary prevention, with the right environment providing opportunity for social connections and personal development (Dumitriu et al., 2025). Evidence is growing around trauma-informed organizational interventions, including staff training (Purtle, 2020). At present, workplaces can be sites of violence (Knight et al., 2022), although International Labor Organization member states have adopted the violence and harassment convention to reduce workplace violence and harassment (Violence and Harassment Convention, 2019). Further research is needed to design and test occupational violence prevention interventions.

## Conclusions

There is a substantial body of evidence that different forms of violence in childhood are associated with a range of negative work outcomes. However, there is a need for data from LMICs as well as on a broader range of outcomes, including child labor. This review re-emphasizes the need for interventions to prevent violence. Preventing violence in the home through parenting interventions, as well as school-based interventions to reduce bullying and sexual violence, are important and will have long-reaching consequences in all aspects of an individual’s work lives, including job aspirations, dismissal, and social mobility.

## Supplemental Material

sj-docx-1-tva-10.1177_15248380251376352 – Supplemental material for How Does Experience of Violence in Childhood Affect Work Outcomes? A Systematic Review of Longitudinal Studies Supplemental material, sj-docx-1-tva-10.1177_15248380251376352 for How Does Experience of Violence in Childhood Affect Work Outcomes? A Systematic Review of Longitudinal Studies by Clare Tanton, Jodie Pearlman, Katherine Merrill, Ligia Kiss, Agnes Kyamulabi, Louise Knight, Fred Kasalirwe, Russell Burke, Eddy Walakira and Karen Devries in Trauma, Violence, & Abuse

sj-docx-2-tva-10.1177_15248380251376352 – Supplemental material for How Does Experience of Violence in Childhood Affect Work Outcomes? A Systematic Review of Longitudinal Studies Supplemental material, sj-docx-2-tva-10.1177_15248380251376352 for How Does Experience of Violence in Childhood Affect Work Outcomes? A Systematic Review of Longitudinal Studies by Clare Tanton, Jodie Pearlman, Katherine Merrill, Ligia Kiss, Agnes Kyamulabi, Louise Knight, Fred Kasalirwe, Russell Burke, Eddy Walakira and Karen Devries in Trauma, Violence, & Abuse

sj-docx-3-tva-10.1177_15248380251376352 – Supplemental material for How Does Experience of Violence in Childhood Affect Work Outcomes? A Systematic Review of Longitudinal Studies Supplemental material, sj-docx-3-tva-10.1177_15248380251376352 for How Does Experience of Violence in Childhood Affect Work Outcomes? A Systematic Review of Longitudinal Studies by Clare Tanton, Jodie Pearlman, Katherine Merrill, Ligia Kiss, Agnes Kyamulabi, Louise Knight, Fred Kasalirwe, Russell Burke, Eddy Walakira and Karen Devries in Trauma, Violence, & Abuse

sj-docx-4-tva-10.1177_15248380251376352 – Supplemental material for How Does Experience of Violence in Childhood Affect Work Outcomes? A Systematic Review of Longitudinal Studies Supplemental material, sj-docx-4-tva-10.1177_15248380251376352 for How Does Experience of Violence in Childhood Affect Work Outcomes? A Systematic Review of Longitudinal Studies by Clare Tanton, Jodie Pearlman, Katherine Merrill, Ligia Kiss, Agnes Kyamulabi, Louise Knight, Fred Kasalirwe, Russell Burke, Eddy Walakira and Karen Devries in Trauma, Violence, & Abuse
