# Supplementary material for: How Does Experience of Violence in Childhood Affect Work Outcomes? A Systematic Review of Longitudinal Studies
Source: Trauma Violence Abuse. 2025 Nov 28;27(5):2085–109. doi: 10.1177/15248380251376352 (PMC13736292; doi:10.1177/15248380251376352)
Supplement: Supplementary file 1 [file sj-docx-1-tva-10.1177_15248380251376352.docx]

Appendix 1: Search strategy

## Ovid MEDLINE(R) and Epub Ahead of Print, In-Process & Other Non-Indexed Citations, Daily and Versions(R)

| Database name | Medline |
| --- | --- |
| Database platform | Ovid |
| Dates of database coverage | 1946 to August 16, 2019 / Nov 3, 2021 |
| Date searched | 19/08/2019 / 04/11/2021 |
| Searched by | RB |
| Number of hits | 3995 / 4924 |

(Search figures for 2019.)

1	exp Child/ (1843443)

2	Adolescent/ (1950459)

3	exp Infant/ (1104289)

4	puberty/ or menarche/ (17144)

5	Child, Abandoned/ (520)

6	exp Child, Exceptional/ (800)

7	"Child of Impaired Parents"/ (5080)

8	Child, Foster/ (76)

9	Child, Orphaned/ (664)

10	Child, Unwanted/ (294)

11	Child, Adopted/ (50)

12	exp Multiple Birth Offspring/ (27836)

13	Siblings/ (10524)

14	Homeless Youth/ (1218)

15	Minors/ (2513)

16	Disabled Children/ (5921)

17	Students/ [may include adult students] (54038)

18	Young Adult/ [19-24 - too old?] (762687)

19	exp Child Welfare/ (30209)

20	exp Child Health Services/ (23412)

21	Adolescent Health Services/ (5406)

22	Foster Home Care/ (3512)

23	Infant Welfare/ (2737)

24	Pregnancy in Adolescence/ (7711)

25	paternal age/ (1317)

26	Adverse Childhood Experiences/ (239)

27	exp Adolescent Behavior/ (29431)

28	exp Child Behavior/ (21948)

29	Child Behavior Disorders/ (19991)

30	child*.mp. (2343628)

31	paediatric*.mp. (62602)

32	pediatric*.mp. (326090)

33	(neonat* or neo-nat*).mp. (279291)

34	newborn*.mp. (737078)

35	infan*.mp. (1244061)

36	preemie*.mp. (128)

37	(baby or babies).mp. (67849)

38	toddler*.mp. (10268)

39	(tot or tots).mp. (3728)

40	boy*.mp. (147715)

41	girl*.mp. (141937)

42	(schoolage* or (school adj1 age*)).mp. (21468)

43	(preschool* or (pre adj1 school*)).mp. (893941)

44	nursery school*.mp. (1073)

45	kindergarten*.mp. (6235)

46	(nursery or nurseries).mp. (12806)

47	schoolchild*.mp. (13379)

48	youngster*.mp. (2443)

49	minor*.mp. (296034)

50	((school or college) adj3 (pupil* or student*)).mp. (43073)

51	primary school*.mp. (11737)

52	elementary school*.mp. (9470)

53	prepubescen*.mp. (971)

54	puberty.mp. (35222)

55	((secondary or grammar or academy or comprehensive or private or public or middle) adj school*).mp. (22358)

56	pubescent*.mp. (826)

57	adolescen*.mp. (2020897)

58	juvenil*.mp. (89326)

59	underage*.mp. (1687)

60	(preteen* or pre-teen*).mp. (460)

61	(teen or teens or teener).mp. (10257)

62	teenage*.mp. (20499)

63	(youth or youths).mp. (73250)

64	young people*.mp. (26202)

65	young person*.mp. (3324)

66	(highschool* or (high adj1 school*)).mp. (30336)

67	sophomore*.mp. (672)

68	(university adj3 student*).mp. (17959)

69	(kid or kids).mp. (7313)

70	offspring.mp. (68044)

71	sibling*.mp. (52724)

72	(foster adj1 (care or home*)).mp. (4551)

73	(transition adj4 adult*).mp. (4644)

74	emerging adult*.mp. (2223)

75	young adult*.mp. (825581)

76	early adult*.mp. (6864)

77	runaway*.mp. (1719)

78	freshm?n.mp. (2199)

79	infan*.jw. (8649)

80	neonat*.jw. (28076)

81	child*.jw. (150148)

82	pediatric*.jw. (403065)

83	paediatric*.jw. (60709)

84	adolescen*.jw. (42865)

85	youth*.jw. (3432)

86	school*.jw. (17347)

87	(("1" or "2" or "3" or "4" or "5" or "6" or "7" or "8" or "9" or "10" or "11" or "12" or "13" or "14" or "15" or "16" or "17" or "18" or "19") adj (year* old or year* of age)).mp. (271034)

88	(("1" or "2" or "3" or "4" or "5" or "6" or "7" or "8" or "9" or "10" or "11" or "12" or "13" or "14" or "15" or "16" or "17" or "18" or "19" or "20" or "21" or "22" or "23" or "24") adj (month* old or month* of age)).mp. (86160)

89	((one or two or three or four or five or six or seven or eight or nine or ten or eleven or twelve or thirteen or fourteen or fifteen or sixteen or seventeen or eighteen or nineteen) adj (year* old or year* of age)).mp. (32929)

90	((one or two or three or four or five or six or seven or eight or nine or ten or eleven or twelve or thirteen or fourteen or fifteen or sixteen or seventeen or eighteen or nineteen or twenty or twenty-one or twenty-two or twenty-three or twenty-four) adj (month* old or month* of age)).mp. (12228)

91	(age* adj ("1" or "2" or "3" or "4" or "5" or "6" or "7" or "8" or "9" or "10" or "11" or "12" or "13" or "14" or "15" or "16" or "17" or "18" or "19") adj year*).mp. (50163)

92	(age* adj ("1" or "2" or "3" or "4" or "5" or "6" or "7" or "8" or "9" or "10" or "11" or "12" or "13" or "14" or "15" or "16" or "17" or "18" or "19" or "20" or "21" or "22" or "23" or "24") adj month*).mp. (16025)

93	(age* adj (one or two or three or four or five or six or seven or eight or nine or ten or eleven or twelve or thirteen or fourteen or fifteen or sixteen or seventeen or eighteen or nineteen) adj year*).mp. (1694)

94	(age* adj (one or two or three or four or five or six or seven or eight or nine or ten or eleven or twelve or thirteen or fourteen or fifteen or sixteen or seventeen or eighteen or nineteen or twenty or twenty-one or twenty-two or twenty-three or twenty-four) adj month*).mp. (1122)

95	or/1-94 [TOTAL FOR CHILD UP TO 19 YO] (5101181)

96	domestic violence/ or spouse abuse/ (12772)

97	Intimate Partner Violence/ (1833)

98	Physical Abuse/ (442)

99	sex offenses/ (8336)

100	rape/ (6156)

101	violence/ (29552)

102	gender-based violence/ (128)

103	gun violence/ (71)

104	physical abuse/ (442)

105	torture/ (2079)

106	workplace violence/ (751)

107	Incest/ (1626)

108	exp Bullying/ (3534)

109	exp Aggression/ (36544)

110	Punishment/ (5363)

111	Crime Victims/ (8676)

112	Sexual Harassment/ (1613)

113	(rape or rapes or raped or raping).ti,ab. (7563)

114	(rapist* or raper*).ti,ab. (551)

115	(sex* adj3 (violen* or abus* or assault* or attack* or aggressi* or coerc* or maltreat* or victim* or re-victim* or offen* or harass* or exploit*)).ti,ab. (29771)

116	(emotion* adj3 (violen* or abus* or assault* or attack* or aggressi* or coerc* or maltreat* or victim* or re-victim* or offen* or harass* or exploit*)).ti,ab. (4303)

117	(psychologic* adj3 (violen* or abus* or assault* or attack* or aggressi* or coerc* or maltreat* or victim* or re-victim* or offen* or harass* or exploit*)).ti,ab. (3452)

118	(physic* adj3 (violen* or abus* or assault* or attack* or aggressi* or coerc* or maltreat* or victim* or re-victim* or offen* or harass* or exploit*)).ti,ab. (17064)

119	(mental* adj3 (violen* or abus* or assault* or attack* or aggressi* or coerc* or maltreat* or victim* or re-victim* or offen* or harass* or exploit*)).ti,ab. (5059)

120	(gender* adj3 (violen* or abus* or assault* or attack* or aggressi* or coerc* or maltreat* or victim* or re-victim* or offen* or harass* or exploit*)).ti,ab. (2981)

121	(without consent or unconsent* or non-consensual or nonconsensual).ti,ab. (885)

122	molest*.ti,ab. (1231)

123	(family adj3 (violen* or abus* or assault* or attack* or aggressi* or coerc* or maltreat* or victim* or re-victim* or offen* or harass* or exploit*)).ti,ab. (4219)

124	((spous* or wife or wives or husband* or partner* or peer* or boyfriend* or boy-friend* or girlfriend* or girl-friend* or acquaintance* or non-stranger* or nonstranger* or relationship* or date or dating) adj3 (violen* or abus* or assault* or attack* or aggressi* or coerc* or maltreat* or victim* or re-victim* or offen* or harass* or exploit*)).ti,ab. (18461)

125	(verbal* adj3 abus*).ti,ab. (1080)

126	(family adj3 crime*).ti,ab. (65)

127	(domestic adj3 (violen* or abus* or assault* or attack* or aggressi* or coerc* or maltreat* or victim* or re-victim* or offen* or harass* or exploit*)).ti,ab. (6327)

128	((expose* or exposure*) adj3 (violen* or abus* or assault* or attack* or aggressi* or coerc* or maltreat* or victim* or re-victiim* or offen* or harass* or exploit*)).ti,ab. (6450)

129	(witness* adj3 (violen* or abus* or assault* or attack* or aggressi* or coerc* or maltreat* or victim* or re-victim* or offen* or harass* or exploit*)).ti,ab. (1499)

130	(bully* or bullies or bullied).ti,ab. (5342)

131	((aggression or aggressive*) adj3 behav*).ti,ab. (22140)

132	punish*.ti,ab. (9534)

133	(polyvictim* or poly-victim*).ti,ab. (232)

134	(anti-bully* or antibully*).ti,ab. (237)

135	(cyberbull* or cyber-bull*).ti,ab. (653)

136	(cybervictim* or cyber-victim*).ti,ab. (160)

137	neglect*.ti,ab. (57583)

138	(victim* adj3 crime*).ti,ab. (851)

139	or/96-138 [TOTAL FOR VIOLENCE NARROW TIAB] (213342)

140	exp Child Abuse/ (29247)

141	"Adult Survivors of Child Abuse"/ (2000)

142	Pedophilia/ (914)

143	p?edophil*.ti,ab. (676)

144	Adverse Childhood Experiences/ (239)

145	Juvenile Delinquency/ (8407)

146	(juvenile* adj3 delinquen*).ti,ab. (1208)

147	(adverse adj3 childhood experience*).ti,ab. (1379)

148	(Child* and CSA).ti,ab. (2152)

149	(Child* and CSE).ti,ab. (148)

150	((child* or infan* or adolescent* or baby or babies or boy* or girl*) adj3 (violen* or abus* or assault* or attack* or aggressi* or coerc* or maltreat* or victim* or re-victim* or offen* or harass* or exploit*)).ti,ab. (36729)

151	or/140-150 [TOTAL FOR CHILD VIOLENCE JOINT TERMS] (59433)

152	95 and 139 (91414)

153	151 or 152 [TOTAL FOR CHILDHOOD VIOLENCE] (119155)

154	exp Work/ (60551)

155	Work Capacity Evaluation/ (5841)

156	exp Personnel Management/ (150419)

157	exp psychology, industrial/ (83891)

158	exp Labor Unions/ (8957)

159	exp Employment/ (82457)

160	exp Occupations/ (33507)

161	Women, Working/ (5243)

162	exp Occupational Stress/ (11695)

163	Rehabilitation, Vocational/ (9319)

164	Occupational Therapy/ (12726)

165	Sick Leave/ (5532)

166	Burnout, Professional/ (10746)

167	Occupational Health/ (31999)

168	exp Occupational Diseases/ (128340)

169	exp Income/ (61047)

170	exp Occupational Exposure/ (61081)

171	exp Industry/ (301578)

172	Accidents, Occupational/ (17202)

173	Occupational Injuries/ (2504)

174	Sexual Harassment/ (1613)

175	Workplace Violence/ (751)

176	Workers' Compensation/ (7374)

177	"Compensation and Redress"/ (2923)

178	exp Disability Evaluation/ (50408)

179	caregivers/ (33489)

180	exp occupational groups/ (572959)

181	Volunteers/ (9337)

182	Child Labor/ (9)

183	work*.ti,ab. (1411693)

184	job*.ti,ab. (59145)

185	labo?r.ti,ab. (97603)

186	employ*.ti,ab. (551319)

187	unemploy*.ti,ab. (16549)

188	underemploy*.ti,ab. (334)

189	under-employ*.ti,ab. (80)

190	vocation*.ti,ab. (11260)

191	occupation*.ti,ab. (147913)

192	career*.ti,ab. (38463)

193	welfare.ti,ab. (33787)

194	presentee*.ti,ab. (1188)

195	absentee*.ti,ab. (5622)

196	((sick* or ill*) adj3 (leave or absen*)).ti,ab. (8218)

197	(attendance adj2 record*).ti,ab. (383)

198	earning*.ti,ab. (5144)

199	wage*.ti,ab. (7397)

200	work-related.ti,ab. (14917)

201	industr*.ti,ab. (240738)

202	Sex Work/ (5878)

203	Sex Worker/ (1836)

204	prostitut*.ti,ab. [requested by researchers] (3674)

205	Human Trafficking/ (340)

206	Enslavement/ (90)

207	Enslaved Persons/ (48)

208	(trafficking or trafficked).ti,ab. (52452)

209	slave*.ti,ab. (2672)

210	enslave*.ti,ab. (243)

211	servitude.ti,ab. (73)

212	modern-slave*.ti,ab. (27)

213	exploit*.ti,ab. (114095)

214	or/154-213 [TOTAL WORK OUTCOMES] (3301501)

215	153 and 214 [TOTAL FOR CHILDHOOD VIOLENCE AND WORK OUTCOMES] (24331)

216	Case-Control Studies/ or Control Groups/ or Matched-Pair Analysis/ or prospective studies/ (763678)

217	((case* adj5 control*) or (case adj3 (comparison* or base or refer?ent*))).ti,ab,kw. (185326)

218	control-group*.ti,ab,kw. (429848)

219	(matched-pair adj3 analys?s).ti,ab,kw. (1174)

220	(paired adj3 comparison*).ti,ab,kw. (3045)

221	prospective studies/ or prospective.ti,ab,kw. (748652)

222	or/216-221 (1430171)

223	221 and 222 [CASE CONTROL STUDIES PROSPECTIVE ONLY] (748652)

224	cohort studies/ or longitudinal studies/ or follow-up studies/ or prospective studies/ (1314229)

225	(cohort or longitudinal or prospective).ti,ab,kw. (1091368)

226	((concurrent or incidence or follow-up or followup) adj3 stud*).ti,ab,kw. (90282)

227	224 or 225 or 226 [COHORT STUDIES] (1902741)

228	("clinical trial" or "clinical trial, phase i" or "clinical trial, phase ii" or clinical trial, phase iii or

clinical trial, phase iv or controlled clinical trial or "multicenter study" or "randomized controlled trial").pt. or double-blind method/ or clinical trials as topic/ or clinical trials, phase i as topic/ or clinical trials, phase ii as topic/ or clinical trials, phase iii as topic/ or clinical trials, phase iv as topic/ or controlled clinical trials as topic/ or randomized controlled trials as topic/ or early termination of clinical trials as topic/ or multicenter studies as topic/ or ((randomi?ed adj7 trial*) or (controlled adj3 trial*) or (clinical adj2 trial*) or ((single or doubl* or tripl* or treb*) and (blind* or mask*))).ti,ab,kw. or ("4 arm" or "four arm").ti,ab,kw. [CLINICAL TRIALS] (1553511)

229	215 and 223 (823)

230	215 and 227 (2957)

231	215 and 228 (1333)

232	229 or 230 or 231 (3995)
