# Supplementary material for: How Does Experience of Violence in Childhood Affect Work Outcomes? A Systematic Review of Longitudinal Studies
Source: Trauma Violence Abuse. 2025 Nov 28;27(5):2085–109. doi: 10.1177/15248380251376352 (PMC13736292; doi:10.1177/15248380251376352)
Supplement: Supplementary file 2 [file sj-docx-2-tva-10.1177_15248380251376352.docx]

**Appendix 2: Detailed descriptives of included studies**

| **Dataset drawn from** | **Study location** | **Study population** | **Age (year) of enrolment** | **Sample size of original cohort** | **Sample size of study population** | **% followed-up** | **Length of follow-up** | **Violence measures** | **Age(s) when violence measures collected** | **Work measures** | **Age(s) when work measures collected** | **Quality assess-ment score of report** | **Author (year)** |
| --- | --- | --- | --- | --- | --- | --- | --- | --- | --- | --- | --- | --- | --- |
| Avon Longitudinal Study of Parents and Children (ALSPAC) | Avon, England | Children of pregnant women resident in Avon with an estimated due date between 1 April 1991 and 31 December 1992 | Birth | 15,589 | 14,694 | 27.2% | 12 yrs | 1) Bullying (self-report)  2) Emotional abuse (parent-report)  3) Emotional neglect (self-report)  4) Physical abuse (parent-report)  5) Violence between child and partner (self-report) | 11-16 yrs | NEET (self-report) | 23 yrs | 6.0 | Cahill et al. (2022) |
|  |  |  |  |  | 15,589 | 40.0% | 25 yrs | Between 0-16 years:  1) Any child maltreatment (no parental DVA) (self- and parent-report)  2) Parental DVA (no child maltreatment) (parent-report)  3) Parental DVA (child aware) (self- and parent-report)  4) Any child maltreatment and/or parental DVA (self- and parent-report)  5) Any child maltreatment and/or parental DVA (child aware) (self- and parent-report)  6) Emotional abuse and parental DVA (self- and parent-report)  7) Emotional abuse and physical abuse and parental DVA (self- and parent-report)  8) Any child maltreatment and parental DVA (self- and parent-report)  9) Any child maltreatment and parental DVA (child aware) (self- and parent-report) | 0-22 years (combination of prospective and retrospective) | Mean earnings loss (self-report) | 25 yrs | 5.5 | Herbert et al. (2023) |
|  |  |  |  |  | 2,710 | 18.2% | 28 years | 1) Physical abuse by adult in family ever (self- and parent- report)  2) Sexual abuse ever (self- and parent-report)  3) Emotional abuse by parent ever (self- and parent-report)  4) Emotional neglect (self- and parent-report)  5) Bullying on weekly basis (self-report) | 0-23 years | 1) Change in employment since pandemic (self-report)  2) Claimed benefits since pandemic (self-report) | Mean age 27.8 years (May-July 2020) | 6.5 | Smith et al. (2022) |
| Cape Area Panel Study (CAPS) | Cape Town, South Africa | Youth and young adults undergoing the multiple transitions from adolescence to adulthood in Cape Town, South Africa | 14-22 yrs (2002) | 4,752 | 4,752 | 55.6% | 7 yrs | 1) Child maltreatment (physical and/or emotional abuse) up to 14 yrs (self-report) 2) Physical abuse (self-report) 3) Emotional abuse up to 14 yrs (self-report) | 14-22 yrs (i.e. once at enrolment) | 1) Employment (assumed self-report) 2) Typical monthly wages (in South African Rands) (self-report) | 21-29 yrs | 6.5 | Zheng et al. (2018) |
| Christchurch Health and Development Study | Christchurch, New Zealand | Children born in the Christchurch urban region over a 4-month period in 1977 | Birth (1997) | 1,265 | 1,265 | 77.8% | 30 yrs | Sexual abuse prior to age 16 (self-report) | 18 and 21 yrs | 1) Welfare dependence (self-report) 2) Income in past year (self-report) | 1) 25-30 yrs 2) 30 yrs | 5.5 | Fergusson et al. (2013) |
|  |  |  |  |  | 1,265 | 74-80% | 30 yrs | 1) Sexual abuse prior to 16 yrs (self-report) 2) Physical abuse by parent prior to 16 yrs (self-report) | 18 and 21 yrs | 1) Welfare dependence (self-report) 2) Income in past year (self-report) 3) Economic hardship (self-report) 4) Working in paid employment (self-report) | 30 yrs | 6.5 | Gibb et al. (2012) |
| Chicago Longitudinal Study | Chicago, United States | Low-income minority children born in 1979 or 1980 who grew up in high-poverty neighbourhoods and attended government-funded early childhood programmes in the Chicago Public Schools | 3-5 yrs (1979-80) | 1,539 | 1,410 | N/A | 25 yrs | Child abuse and neglect (child protection service records) | Kindergarten - 18 yrs | Occupational prestige (self report where available, otherwise administrative data) | 22-24 yrs | 8.0 | Herbers et al. (2013) |
|  |  |  |  |  | 1,142 | 77.5-77.9% | 30-34 yrs | Child abuse and neglect (administrative records) | Up until 18 yrs | Income (more or less than the average wage) (administrative records from 2010-14 where available, otherwise self-report) | 35-37 yrs | 7.0 | Giovanelli et al. (2018) |
| Environmental Risk (E-Risk) Longitudinal Twin Study | England and Wales | Birth cohort of same-sex twins born in 1994 to 1995 | 5 yrs (1999-2000) | 2,232 | 2,232 | 93.0% | 13 yrs | Physical/sexual maltreatment by an adult (mother-report) | 5, 7, 10, and 12 yrs | NEET (self-reported) | 18 yrs | 7.0 | Jaffee et al. (2018) |
| The Great Smoky Mountain Study | North West Carolina, United States | Children aged 9, 11 and 13 recruited from 11 counties in western North Carolina | 9, 11 and 13 yrs (1993) | 1,420 | 1,420 | 89.6% | 10-11 yrs | Being bullied (self- and parent-report) | Annually from 9-16 yrs | 1) Poverty (self-report) 2) Dismissal from a job (self-report) 3) Quitting multiple jobs (self-report) | 19, 21, 24-26 yrs | 6.0 | Wolke et al. (2013) |
| The Lehigh Longitudinal Study | Eastern Pennsylvania, United States | Children from two child welfare groups (substantiated abuse or neglect) or other settings, including Head Start, day care and middle-income nursery | 18 months - 6 yrs (1976) | 457 | 457 | 78.1% | 32-34 yrs | Maltreatment (previous reports to child protection services) | 18 months - 6 yrs | 1) Unemployment problems in past year (self-report) 2) Income problems (composite measure with different timepoints, self-report) | 31-41 yrs 31-41 yrs | 6.5 | Stevens et al. (2018) |
| Mater-University of Queensland Study of Pregnancy (MUSP) | Queensland, Australia | Children of pregnant women who attended the Mater Misericordiae Mothers' Hospital for their first prenatal visit. Multiple births, adoptions, perinatal deaths were excluded. | Birth (1981) | 7,223 | 7,223 | 52.3% | 21 yrs | 1) Notified maltreatment (abuse and/or neglect) 2) Substantiated maltreatment (abuse and/or neglect) (from records of the state child protection agency) | Before aged 16 yrs | Currently not in education or employment (self-report) | 21 yrs | 6.0 | Mills et al. (2019) |
| National Longitudinal Survey of Youth 1997 (NLSY) | United States | Nationally representative sample of youth | 12-16 yrs (1997) | 8,984 | 8,901 | Not specified | 14 yrs | Childhood victimisation (victim of violent crime, bullying, gun violence, perceived school safety, threatened violence at school) (self-report) | Perceived school safety, threatened violence: 12-16 yrs (though analysis uses follow-up from 1999-2009)  Bullying and gun violence, victim of violent crime (retrospective): ≥ 18 yrs | 1) Occupational prestige in past year (pooled time; self-report) 2) Annual income in past year (pooled time; self-report) 3) Occupational prestige in past year (baseline) 4) Annual income in past year (baseline; self-report) 5) Trajectory of occupational prestige in past year (self-report) 6) Trajectory of annual income in past year (self-report) | Annually from 14-18 to 24-28 yrs | 6.5 | Fernandez et al. (2015) |
|  |  |  | Calculated as 13-17 yrs (1997) |  | Not specified | Not specified | 9 yrs | 1) Bullied under 12 yrs (self-report) 2) Bullied teen (self-report) | Not clear. Suggests 13-17 yrs and after 18 yrs | Income in 2006 (self-report) | 22-26 yrs | 5.0 | Mukerjee et al. (2018) |
| National Longitudinal Study of Adolescent to Adult Health (ADD Health) | United States | Junior high, middle school and high school students | 13-17 yrs (1994-1995) | 20,745 | 1,386 | 100.0% | Not specified | Emotional/physical IPV (self-report) | 13-17 yrs | Current employment status (self-report) | 24-32 yrs | 5.0 | Heng (2015) |
| National Youth Survey Family Study (NYSFS) | United States | Individuals in the US who were 11-17 yrs | 11-17 yrs (1976-77) | 1,725 | 1,725 | 75% in Wave 10 and 70% in Wave 11 (2002-3) | 27 yrs | 1) Ever witnessing parental violence (self-report) 2) Physical abuse in past year (self-report) | 1) 24-30 through 37-43 yrs 2) 11-17 through 15-21 yrs | 1) Current employment (self report) 2) Income in past year (self-report) | 1) 37-43 yrs  1) 37-43 yrs | 4.5 | Covey et al. (2013) |
|  |  |  |  |  | 1,725 | 82.0% | 10 yrs | 1) Adolescent victimisation: Combined measure of sexual assault, physical beating and attacked with weapon (self-report) 2) Sexual assault in the past year (self-report) 3) Physical beating in past year, not by parent (self-report) 4) Attacked with weapon in past year, not by parent (self-report) | Collected one-year incidence measures at each of 7 waves of data collection | Income deficits in later adulthood for 1986 (self-report) | 21-27 yrs | 7.0 | Macmillan (2000) |
|  |  |  |  |  | 1,725 | 1983: 82% 1986: Not specified | 10 yrs | Adolescent victimisation (beat up, robberies, sexual assaults, attacked with weapon) before the age of 18 (self-report) | Collected one-year incidence measures at each of 7 waves of data collection | 1) Current occupational status (self-report) 2) Labour force participation in past year (self-report) 3) Unemployed in past year (self-report) 4) On public assistance in 1986 (self-report) 5) Average hourly wage in 1986 (self-report) | 21-27 yrs | 6.0 | Macmillan (1998) |
|  |  |  |  |  | 1,725 | 80.0% | 10 yrs | Log of the average annual incidence of adolescent victimisation (beat up, attacked with weapon or sexually assualted) before the age of 18 (self-report) | Between ages 13 and 17, specific yrs not clear | 1) Current occupational status (self-report) 2) Labour force participation in past year (self-report) 3) Unemployed in past year (self-report) 4) On public assistance in 1986 (self-report) 5) Average hourly wage in 1986 (self-report) | 21-27 yrs | 6.5 | Macmillan and Hagan (2004) |
| National Child Development Study (NCDS), the 1958 British birth cohort | Great Britain | Individuals born in one week in March 1958 across England, Scotland, and Wales | Birth (1958) | 18,558 | 18,558 | 43.9% | 50 yrs | 1) Neglect (parent- and teacher-report) 2) Emotional neglect (self-report) 3) Sexual abuse by parent (self-report) 4) Physical abuse by parent (self-report) 5) Psychological abuse by parent (self-report) 6) Witnessing IPV of others in family (self-report) 7) Non-sexual abuse (physical abuse, psychological abuse, witnessing IPV) (self-report) 8) Number of maltreatment types (includes non-sexual abuse, neglect and emotional neglect; self-, parent-and teacher-report) | 1) 7 and 11 yrs 2) 45 yrs 3) 45 yrs 4) 45 yrs 5) 45 yrs 6) 45 yrs 7) 45 yrs 8) 7, 11 and 45 yrs | 1) Long-term sickness absence (self-report) 2) NEET (self-report) 3) Current income-related support (self-report) 4) Manual occupation (self-report and parent-report)† 5) Intergenerational social mobility (self-report and parent-report) 6) Intragenerational social mobility (self-report) | 1) Sickness absence at 23 yrs, permanently sick/disabled at 50 yrs 2) 23 and 50 yrs 3) 50 yrs 4) 23 and 50 yrs 5) 50 yrs 6) 23 and 50 yrs | 5.0 | Pereira et al. (2016) |
|  |  |  |  |  | 18,558 | Net individual earnings: 37.1%  Welfare dependence: 40.1% | 55 yrs | Physical neglect (teacher-reported) | 7 and 11 yrs | 1) Net individual earnings for year 2011 (self-report) 2) Welfare dependence (self-report) | 55 yrs | 6.0 | Schurer et al. (2019) |
|  |  |  |  |  | 18,558 | 49.8% | 50 yrs | Bullying (parent-report) | 7 and 11 yrs | 1) Current economic status (self-report) 2) Current earnings (self-report) | 50 yrs | 5.5 | Brimblecombe et al. (2018) |
|  |  |  |  |  | 18,558 | 49.2% | 55 yrs | 1) Neglected appearance (medical examiner-reported) 2) Physical/sexual abuse from 0-16 yrs (self-report) | 1) 7 and 11 yrs 2) 45 yrs | Current labour force participation (part-time work, unemployed, permanent sick, retired, homemaker/other) (self-report) | 55 yrs | 4.5 | Fahy et al. (2017) |
|  |  |  |  |  | 18,558 | 59.2% | 42 yrs | Bullying (mother-reported) | 7 and 11 yrs | Wages | 23, 33 or 42 yrs | 4.0 | Brown et al. (2008) |
|  |  |  |  |  | ~9,400 | 50 yrs: ~74.9% 55 yrs: ~64.5% | 55 yrs | Child maltreatment: experience of neglect by mother, father or anyone else, or abuse (physical, emotional, sexual) (self-report) | Before aged 16 yrs | 1) Employed (self-report) 2) Net weekly earnings if employed (self-report) | 50 and 55 yrs | 4.0 | Conti et al. (2021) |
|  |  |  |  |  | 18,558 | 61.0% | 50 yrs | Bullying (parent-report) | 7 and 11 yrs | 1) Current unemployment (self-report) 2) Current weekly net pay (self-report) | 50 yrs | 6.0 | Takizawa et al. (2014) |
|  |  |  |  |  | 18,558 | 23 yrs: 59.1%  33 yrs: 53.7%  42 yrs: 53.8%  46 yrs: 45.2%  50 yrs: 46.2%  55 yrs: 41.7% | 55 yrs | Being bullied sometimes or frequently (parent-report) | 7 and 11 yrs | Being in paid work | 23, 33, 42, 46, 50, 55 yrs | 4.0 | Blanchflower et al. (2024) |
| Next Steps | England | English children born in 1989/90. Children are representative of the English population but specific groups were oversampled, including those youths from low socioeconomic backgrounds and ethnic minorities | 13/14 yrs (2004) | 15,500 | ~15,000 | Not specified at age 25 yrs (~25% missing at Wave 1; ~10% missing in each subsequent wave (from ages 14-21), with further decrease at age 25 | 11-12 yrs | Being bullied in the past year (self-report) | 13-16 yrs (3 waves) | 1) Weekly income (self-report) 2) Unemployment (self-report) | 25 yrs | 6.5 | Gorman et al. (2021) |
|  |  |  |  |  | 15,500 | 47.3% | 6 yrs | Experience of aggressive behaviour in school in the past year (self-report) | 13 yrs | NEET for ≥6 months in 2006-9 (self-report) | 16-19 yrs (measured annually over 3 waves) | 7.0 | Karyda (2020) |
|  |  | English children born in 1989/90. Children are representative of the English population but specific groups were oversampled, including those youths from low socioeconomic backgrounds and ethnic minorities. Only among individuals with common mental health conditions. |  |  | 2,224 | 83.1% | 10 yrs | Being bullied since start of study (approx past year; self-report) | 15-16 yrs (wave 2) | Current NEET (self-report) | 25-26 yrs (wave 8) | 7.0 | Tayfur et al. (2022) |
| Quebec Longitudinal Study of Kindergarten Children (QLSKC) | Quebec, Canada | Children born in 1980 or 81 and recruited in kindergarten in French-language public schools in Quebec, Canada, during the 1986−1987 and 1987−1988 school years. 2000 children were representative of Quebec kindergarten schoolchildren (randomly sampled) and 1,020 were oversampled for disruptive behaviour. | 6 yrs | 3,020 | 3,020 | Unclear at final follow-up | 31 yrs | 1) Any child sexual abuse (official report, self-report) 2) Type of child sexual abuse - extrafamilial vs intrafamilial (self-report) 3) Severity of child sexual abuse - contact vs no contact (self-report) 4) Chronicity - multiple episodes vs single episode (self-report) | Official reports: before age 18 years  Self-report: retrospectively at 22 years | Average employment earnings, USD (government tax records) | 33-37 yrs | 6.0 | Bouchard et al. (2023) |
|  |  |  |  |  | 3,020 | 56.0% | 31 yrs | Physical and/or sexual abuse (self-report) | 22 yrs | Total number of years receiving social welfare benefits between ages 23-37 years (government tax records) | 23-37 yrs | 6.0 | Domond et al. (2023) |
| Simmons Longitudinal Study | New England, United States | Children entering kindergarten within a single public school system participating in state-mandated preschool testing of developmental, academic, and behavioural factors | 5 yrs (1977) | 519 | 519 | 66.9% | 15 yrs | Family physical violence to participant or another family member (self-report) | 18 yrs | 1) Current unemployment (self-report) 2) Lack of long-range career goals (self-report) | 30 yrs | 4.5 | Paradis et al. (2009) |
| Study of Alameda County Homeless Residents (STAR) Project | Alameda County, California, United States | Homeless adults who were at least 18 yrs old | At least 18 yrs old (1991) | 564 | 564 | 70.4% | 15 months | Physical or sexual abuse under the age of 18 yrs, collected retrospectively (self-report) | ≥ 18 yrs (baseline) | Labour force participation for past four weeks (self-report) | ≥18 yrs (15 months post-baseline) | 5.0 | Tam et al. (2003) |
| Taiwan Youth Project Surveys | Taipei city, Taipei county, and Yi-Lan county, Taiwan | School-based representative samples of junior high students in Taipei city, Taipei county, and Yi-Lan county | Two cohorts: 1) J1, Grade 7: average=13 yrs (2000) 2) J3, Grade 9: average=15 yrs (2000) | 5,541 | 5,541 | 52.4% | 1) J1: 7 yrs 2) J3: 5 yrs | 1) Abuse (physical and emotional) during childhood (self-report) 2) Emotional neglect by parent during childhood (self-report) | 1) J1, Grade 7: average=13 yrs 2) J3, Grade 9: average=15 yrs | NEET (current) (self-report) | Mean is 20 yrs (sophomore year) | 5.0 | Lin and Chiao (2021) |
| Toronto Urban Youth Project | Toronto, Canada | Individuals from all four secondary schools in the central area of the community | 12-21 yrs (1976) | 834 | 834 | 81.7% | 13 yrs | Adolescent victimisation before 18 yrs (self-report) | 12-21 yrs | Current occupational status (self-report) | Most between 25 and 32 yrs | 6.0 | Macmillan (1998) |
| TRacking Adolescents’ Individual Lives Survey (TRAILS) | Netherlands | Children born between October 1 1989 and 30 September 1991 in three northern provinces of Netherlands | Birth | 2,229 | 2,229 | 68.4% | 22 yrs | 1) Sexual abuse by family member, family acquaintance or stranger between 0-16 years (self-report)  2) Physical abuse by parent between 0-16 years (self-report)  3) Emotional abuse by parent between 0-16 years (self-report) | 16 yrs | NEET (derived from current educational status, past month employment status, current benefit receipt, current work hours/week and monthly income) (self-report) | 22 yrs | 5.0 | De Vries et al. (2023) |
| Wisconsin Administrative Data Core | Wisconsin, United States | Children born in 1993-1996 who resided in Milwaukee County at some point during chilldhood, and who had electronic child welfare records beginning in January 2000 | Mostly 3-7 yrs (2000) | 29,154 | 29,154 | 98.5% | 13-17 yrs | Maltreatment (administrative records from child welfare system) | From year 2000 until 16th birthday | 1) Stable employment (i.e. employed 75-100% of time) during ages 18 and 19 (self-report) 2) Average quarterly earnings (obtained from Unemployment Insurance system) | Before aged 20 (for ages 18 and 19 yrs) | 8.0 | Font and Maguire-Jack (2020) |
| Women’s Employment Study | Michigan, United States | Single mothers receiving welfare in one county in Michigan | 18-54 yrs (1997) | 753 | 753 | 71.2% | ~6-7 yrs | Adolescent IPV before age 18 (self-report) | 18-54 yrs | 1) Adult earnings from employment in past year (self-report) 2) Growth in earnings over time in past year (self-report) ‡ | T1: 19-55 yrs T2: 21-57 yrs T3: 23-59 yrs | 5.0 | Adams (2013) |
| Youth and Mental Health Study | South- and North-Trøndelag, Norway | Representative sample of students | 12-15 yrs (1998) | 2,464 | 2,532 | 51.9% | 13-14 yrs | Bullying involvement in past 6 months (self-report) | 12-15 yrs and 13-17 yrs (one year later) | 1) Current occupation status: disability/social help/unemployed (self-report) 2) Current occupation status: working full-time/part-time (self-report) | 26-28 yrs | 4.0 | Sigurdson et al. (2014) |
| The Youth Studies | Norway | All the tenth graders in six participating counties: Oslo, Hedmark, Oppland, Nordland, Troms, and Finmark) | 15-16 yrs (1999-2004) | 15,966 | 14,063 | 84.4% | 5-8 yrs | Polyvictimisation from sexual abuse, physical violence and being bullied in past year (self-report) | 15-16 yrs | Work participation (registry data) | 21-24 yrs | 7.0 | Strom et al. (2013) |
| Zurich Project on the Social Development from Childhood into Adulthood (z-proso) | Zurich, Switzerland | All 2,520 children who entered the first grade of school in Zurich, Switzerland, in 2004 | 7.45 yrs (SD=0.39) | 1,675 | 1,675 | Wave 6 (age 15): 86% Wave 7 (age 17): 78% | 10 yrs | 1) Violence victimisation in past year (robbery, serious assault with a weapon, serious assault without a weapon but with injury, sexual assault, simple assault, sexual harassment) (self-report) 2) Corporal parental punishment (current) (self-report) | 15 yrs | Selling sexual services in past two yrs (self-report) | 17 yrs | 5.0 | Averdijk et al., (2020) |
| Not named (Widom et al. 1989) | Midwestern metropolitan county area, United States | Children with court substantiated cases of childhood physical and sexual abuse and neglect processed from 1967 to 1971 | ≤11 yrs (1967-71) | 1,575 | 1,196 | 67.5% | 37 yrs | Child abuse and neglect (court-substantiated cases) | ≤11 yrs | 1) Manual occupation (self-report)† 2) Employment status (self-report) 3) Imputed earnings (self-report) | 1) 18-40 yrs 2) Mean=41.3 yrs 3) Mean=41.3 yrs | 7.0 | Currie et al. (2010) |
|  |  |  |  |  | 1,196 | Not specified | 22.3 yrs (mean) | Child abuse and neglect (court-substantiated cases) | <11 yrs | 1) Manual occupation (self-report)† 2) Employment status (self-report) | 18-40 yrs | 5.5 | Baetz (2015) |
|  |  |  |  |  | 1,575 | 56.9% | 29-40 yrs | Child abuse and neglect (court-substantiated cases) | ≤11 yrs | Individual-level SES (based on income, receipt of public assistance, unemployment) (self-report) | Mean=40 yrs | 6.0 | Carpi et al. (2020) |
| Not specified | Central Western Ontario, Canada | Extremely low birthweight survivors born between 1977 and 1982, matched  matched on age, sex, and familial SES with a group of control children born at normal birth weight* | 8 yrs | 145 | 145 | 61.0% | 21-28 yrs | Physical or emotional peer victimisation (self-report) | <16 yrs (collected retrospectively at ages 22-26 yrs) | Employed in past year (self-report) | 29-36 yrs | 5.0 | Day et al. (2017) |
| N/A | Ringkjoebing county, Denmark | Individuals born in 1989 in the county of Ringkjoebing, Denmark and still living there in early April 2004 | 14-15 yrs (2004) | N/A | 3681 | 83.0% | 7 yrs | 1) Abuse during lifetime (self-report) 2) Witnessed a very violent event during lifetime (self-report) | 14-15 yrs | Labour market participation from 2010-11 | 21-22 yrs | 7.0 | Lund et al. (2013) |
| *Data extracted refers only to the normal birth weight group  † These outcomes were described by authors using slightly different terminology, but we considered them to be measuring manual occupation  ‡ Growth in earnings is only assessed as an outcome in mediation analyses so is not included in the total count for outcomes | | | | | | | | | | | | | |
