# Supplementary material for: How Does Experience of Violence in Childhood Affect Work Outcomes? A Systematic Review of Longitudinal Studies
Source: Trauma Violence Abuse. 2025 Nov 28;27(5):2085–109. doi: 10.1177/15248380251376352 (PMC13736292; doi:10.1177/15248380251376352)
Supplement: Supplementary file 3 [file sj-docx-3-tva-10.1177_15248380251376352.docx]

**Appendix 3: Results for included studies on the association between violence against children and work outcomes**

| **Exposure** | **Cohort** | **Author (year)** | **Sample (included in analysis)** | **% or mean(SD) reporting violence** | **Outcome** | **% or mean(SD) of exposed group reporting work outcome** | **Crude effect estimate** | **Adjusted effect estimate** | **Variables controlled for (if applicable)** |
| --- | --- | --- | --- | --- | --- | --- | --- | --- | --- |
| ***Child abuse and neglect (official records from child welfare agencies/court cases)*** | | | | | | | | | |
| Child abuse/neglect | Chicago Longitudinal Study | Herbers et al. (2013) | 1,410 | 12.2% | Occupational prestige | Not reported | Not reported | Beta (SE)=-0.21 (0.11) (p value>0.05) | School mobility K-12, Residential mobility, gender, ethnicity, CPC preschool, CPC grade school, risk index, K achievement, social maturity, grade retention, special education, juvenile delinquency |
| Child abuse/neglect | Chicago Longitudinal Study | Giovanelli (2018) | 1,228 | 12.2% | Income | Not reported | Not reported | Log OR=0.52 Marginal effect (95% CI)=-0.137 (-0.217--0.057), p=0.001 Standard mean difference=-0.39 | Sex, race, CPC preschool participation, CPC school-age participation, index of family ecology of risk (maternal education at child’s birth, public assistance use, employment status of mother, whether or not the mother was younger than age 18 at childbirth) |
| Child abuse/neglect | The Lehigh Longitudinal Study | Stevens et al. (2018) | Not clear: either 357 or 457 | 54.5% | Unemployment problems | 45.9% | X²=9.81, p=0.002 | Not reported | N/A |
|  |  |  |  |  | Income problems | Not reported | t=0-5.06, p<0.001 | Not reported |  |
| Child abuse/neglect | Not named (Widom et al. 1989) | Baetz (2015) | 1,195 | 56.6% | Unemployed | 54.9% | Not reported | OR (95% CI)=2.56 (1.75-3.74), p<0.001 OR (95% CI)=2.45 (1.67-3.60), p<001* | Race, gender, and age at first interview *Additionally adjusted for self-reported delinquency |
|  |  |  |  |  | Manual occupation (unskilled job)^¶^ | 62.6% | Not reported | OR (95% CI)=2.05 (1.61-2.59), p<0.001 OR (95% CI)=2.05 (1.61-2.60), p<0.001* |  |
| Child abuse/neglect | Not named (Widom et al. 1989) | Currie et al. (2010) | All: 791 Males: 372-447 Females: 344-419 | All: 56-58% at each time period Males: 45.6% Females: 54.4% | Manual occupation (skilled job)^¶^ | All: 37.4% | All: Chi square=21.96, p<0.001 | All: OR=0.66 (95%CI: 0.47-0.91), p<0.01 Males: OR=0.58, 95%CI=0.36-0.96, p<0.05 Females: OR=0.69, 95%CI=0.42-1.13, p value not less than 0.05 | Sex, race/ethnicity, and age, whether the parents ever received welfare or food stamps during the participant’s childhood, whether the participant ever attended Head Start, whether the mother was employed during the participant’s childhood, mother’s highest grade of school completed, and quarter of the year the participant was interviewed |
|  |  |  | All: 796 Males: 374-406 Females: 390-422 | Not reported | Employed | All: 63.2% | All: Chi square=18.54, p<0.001 | All: OR=0.61 (95%CI: 0.42-0.86), p<0.01 Males: OR=0.69, 95%CI=0.37-1.28, p value not less than 0.05 Females: OR=0.53, 95%CI=0.34-0.84, p<0.01 |  |
|  |  |  | All: 755 Males: 355-385 Females: 370-400 | Not reported | Imputed earnings ($) | All: $18,888 ($1,055) | All: t=5.16, p<0.001 | All: Coefficient=-5,108 (SE=1,805), p<0.01 Males: Coefficient= -5,060, SE= 3,125, p value not less than 0.05 Females: Coefficient= -5,584, SE= 1,926, p<0.01 |  |
| Child neglect | Not named (Widom et al. 1989) | Currie et al. (2010) | 704 | 56-58% at each time point | Manual occupation (skilled job)^¶^ | Not reported | Not reported | OR (95% CI)=0.64 (0.45-0.91), p<0.05 | Sex, race/ethnicity, and age, whether the parents ever received welfare or food stamps during the participant’s childhood, whether the participant ever attended Head Start, whether the mother was employed during the participant’s childhood, mother’s highest grade of school completed, and quarter of the year the participant was interviewed |
|  |  |  | 709 | 56-58% at each time point | Employed | Not reported | Not reported | OR (95 CI%)=0.59 (0.40-0.86), p<0.01 |  |
|  |  |  | 674 | 56-58% at each time point | Imputed earnings ($) | Not reported | Not reported | Coefficient (SE)=-7,005 (1,805), p<0.001 |  |
| Child abuse/neglect | Not named (Widom et al. 1989) | Carpi et al. (2020) | 631 | 55.6% | Lower individual level SES (based on income, receipt of public assistance, unemployment) | Household income: 36.5% Public assistance: 29.3% Unemployed: 21.1% | Not reported | Beta (SE)=0.27 (0.04); 95% CI=0.18-0.35, p<0.01 | Age, sex, race |
| Alleged neglect | Wisconsin Administrative Data Core | Font and Maguire-Jack (2020) | 28,701 | 10.5% | Stable employment | 29.6% | Not reported | Beta (SE)=-0.308 (0.045), p<0.001 | Year of birth, sex, race/ethnicity, receipt of SSI (as a proxy for disability status), mother’s age at first birth, number of children born to mother, number of fathers to mother’s children, marital status at child’s birth, and subjects’ mothers’ receipt of cash assistance, employment, earnings, and incarceration when the subject was ages 0–5. |
|  |  |  |  |  | Average quarterly earnings | Mean (SD)=$930.18 (1226.56) | Not reported | Beta (SE)=-107.709 (23.621), p<0.001 |  |
| Alleged neglect and abuse | Wisconsin Administrative Data Core | Font and Maguire-Jack (2020) | 28,701 | 7.7% | Stable employment | 30.7% | Not reported | Beta (SE)=-0.338 (0.052), p<0.001 | Year of birth, sex, race/ethnicity, receipt of SSI (as a proxy for disability status), mother’s age at first birth, number of children born to mother, number of fathers to mother’s children, marital status at child’s birth, and subjects’ mothers’ receipt of cash assistance, employment, earnings, and incarceration when the subject was ages 0–5. |
|  |  |  |  |  | Average quarterly earnings | Mean (SD)=$914.19 (1200.99) | Not reported | Beta (SE)=-160.404 (27.800), p<0.001 |  |
| Alleged abuse | Wisconsin Administrative Data Core | Font and Maguire-Jack (2020) | 28,701 | 13.6% | Stable employment | 35.5% | Not reported | Beta (SE)=-0.186 (0.039), p<0.001 | Year of birth, sex, race/ethnicity, receipt of SSI (as a proxy for disability status), mother’s age at first birth, number of children born to mother, number of fathers to mother’s children, marital status at child’s birth, and subjects’ mothers’ receipt of cash assistance, employment, earnings, and incarceration when the subject was ages 0–5. |
|  |  |  |  |  | Average quarterly earnings | Mean (SD)=$1035.01 (1243.87) | Not reported | Beta (SE)=-106.389 (21.829), p<0.001 |  |
| Any notified maltreatment | Mater-University of Queensland Study of Pregnancy (MUSP) | Mills et al. (2019) | 3,739 | 7.6% | Not in education or employment | 31.90% | OR (95% CI)=3.01 (2.22-4.08), p<0.001 | OR (95 CI%)=2.95 (2.17-4.01), p<0.001 | Age, sex |
| Notified child abuse | Mater-University of Queensland Study of Pregnancy (MUSP) | Mills et al. (2019) | 3,739 | 6.5% | Not in education or employment | 31.70% | OR (95% CI)=2.96 (2.14-4.10), p<0.001 | OR (95 CI%)=2.87 (2.07-3.98), p<0.001 | Age, sex |
| Notified child neglect | Mater-University of Queensland Study of Pregnancy (MUSP) | Mills et al. (2019) | 3,739 | 4.0% | Not in education or employment | 32.40% | OR (95% CI)=2.92 (1.93-4.42), p<0.001 | OR (95 CI%)=2.92 (1.92-4.44), p<0.001 | Age, sex |
| Any substantiated maltreatment | Mater-University of Queensland Study of Pregnancy (MUSP) | Mills et al. (2019) | 3,739 | 4.6% | Not in education or employment | 31.00% | OR (95% CI)=2.83 (1.93-4.15), p<0.001 | OR (95 CI%)=2.81 (1.91-4.14), p<0.001 | Age, sex |
| Substantiated child abuse | Mater-University of Queensland Study of Pregnancy (MUSP) | Mills et al. (2019) | 3,739 | 3.9% | Not in education or employment | 30.30% | OR (95% CI)=2.66 (1.75-4.03), p<0.001 | OR (95 CI%)=2.56 (1.68-3.89), p<0.001 | Age, sex |
| Substantiated child neglect | Mater-University of Queensland Study of Pregnancy (MUSP) | Mills et al. (2019) | 3,739 | 2.0% | Not in education or employment | 39.70% | OR (95% CI)=3.86 (2.21-6.75), p<0.001 | OR (95 CI%)=3.84 (2.18-6.77), p<0.001 | Age, sex |
| ***Physical violence*** | | | | | | | | | |
| Physical abuse by parent | National Youth Survey Family Study (NYSFS) | Covey et al. (2013) | Unclear: 1,266 for Wave 10 and 1173 for Wave 11 | 9.6% | Employment | Not reported | r=-0.038, p>0.05 | Beta (SE)=-0.257 (0.356), p=0.470; OR=0.773 | Sex, race/ethnicity, place of residence, family structure, Hollingshead index of social position |
|  |  |  |  |  | Income | Not reported | r=-0.117, p<0.05 | Beta (SE)=-0.608 (0.193), p=0.02 |  |
| Physical abuse by parent | National Child Development Study (NCDS), the 1958 British birth cohort | Pereira et al. (2016) | 8,076 | 5.6% | Long-term sickness absence | Not reported | OR (95% CI)=2.54 (1.78-3.63) § | OR (95% CI)=2.36 (95% CI 1.64-3.40) | Sex, maternal age, birth weight, birth order, poor childhood health, social class in 1958, parental education, household amenities, crowding, and tenure |
|  |  |  |  |  | NEET status | Not reported | OR (95% CI)=1.52 (1.08-2.12) § | OR (95% CI) =1.44 (1.03–2.02) |  |
|  |  |  |  |  | Income-related support | Not reported | OR (95% CI)=1.29 (1.00-1.67) § | OR (95% CI)=1.26 (95% CI 0.98-1.63) |  |
|  |  |  |  |  | Manual occupation^¶^ | Not reported | OR (95% CI)=1.13 (0.90-1.42) § | OR (95% CI)=0.95 (0.75-1.21) |  |
| Physical abuse by parent | Christchurch Health and Development Study | Gibb et al. (2012) | 938-1,009 | Seldom abuse: 78% Regular abuse: 11.2% Frequent/severe abuse: 6.4% | Gross personal annual income | Not reported | Not reported | Not reported. Exposure not significant in the regression model for the effect of childhood family income on life course outcomes | N/A |
|  |  |  |  |  | Welfare dependence | Not reported | Not reported | Not reported. Exposure not significant in the regression model for the effect of childhood family income on life course outcomes | N/A |
|  |  |  |  |  | Economic hardship | Not reported | Not reported | Not reported. Exposure not significant in the regression model for the effect of childhood family income on life course outcomes | N/A |
|  |  |  |  |  | Working in paid employment | Not reported | Not reported | Not reported. Exposure significant in the regression model for the effect of childhood family income on life course outcomes | N/A |
| Physical abuse by parent | TRacking Adolescents’ Individual Lives Survey (TRAILS) | de Vries et al. (2023) | 1,524 | Not reported | NEET | Not reported | Gamma (SE)=0.56 (0.13) | Not reported | N/A |
| Physical abuse by parent/caregiver | Avon Longitudinal Study of Parents and Children (ALSPAC) | Cahill et al. (2022) | 14,964 | 3.2% | NEET | Not reported | Not reported | Not reported | N/A |
| Physical abuse by adult in family | Avon Longitudinal Study of Parents and Children (ALSPAC) | Smith et al. (2022) | 2,707 | 23.0% | Change in employment | Not reported | Ref. same/more hours:  Reduced hours: OR (95% CI)=1.02 (0.70-1.48)  Furlough/paid or unpaid leave: OR (95% CI)=1.30(0.99-1.72)  Stopped working during pandemic: OR (95% CI)=2.15(1.41-3.27)  Not working pre-pandemic: OR (95% CI)=1.43(0.96-2.13) | Ref. same/more hours:  Reduced hours: OR (95% CI)=0.99 (0.67 to 1.45)  Furlough/paid or unpaid leave: OR (95% CI)=1.19 (0.89-1.6)  Stopped working during pandemic: OR (95% CI)=2.32 (1.49-3.6)  Not working pre-pandemic: OR (95% CI)=1.28 (0.83-1.96) | Ethnicity, age, home ownership, mother's marital status, parity, age at participant birth, and educational qualifications of mother and partner |
|  |  |  |  |  | Claimed benefits since pandemic | Not reported | OR (95% CI)=1.15(0.85-1.56) | OR (95% CI)=1.11 (0.81-1.53) | Ethnicity, age, home ownership, mother's marital status, parity, age at participant birth, and educational qualifications of mother and partner |
| Physical violence | Study of Alameda County Homeless Residents (STAR) Project | Tam et al. (2003) | 376 | 12.5% | Labour force participation | 59.6% | p>0.05 | Not reported | N/A |
| Physical beating (not by parent) | National Youth Survey Family Study (NYSFS) | Macmillan (2000) | Not reported | Mean=0.13 (SD=0.33) | Income deficits in later adulthood | Not reported | Not reported | Unstandardised coefficient(SE) = -1.13 (0.36), p<0.001 | Gender, age at entry, family SES in adolescence, race, intact family, urban residence, suburban residence, delinquent peers, total delinquency |
| Attacked with weapon | National Youth Survey Family Study (NYSFS) | Macmillan (2000) | Not reported | Mean=0.16 (SD=0.37) | Income deficits in later adulthood | Not reported | Not reported | Unstandardised coefficient(SE) = -0.69 (0.33), p<0.001 | Gender, age at entry, family SES in adolescence, race, intact family, urban residence, suburban residence, delinquent peers, total delinquency |
| Physical abuse ("push" and/or "hit hard") | Cape Area Panel Study | Zheng et al. (2018) | All: 2,644 Male: 1,197 Female: 1,477 | All: 34% Male: 34% Female: 34% | Typical monthly wages (in South African Rands) | Not reported | Not reported | All: Coefficient (SE)=-0.14 (0.03), p<0.01 Male: Coefficient (SE)=-0.08 (0.05), p<0.1 Female: Coefficient (SE)=-0.20 (0.05), p<0.01 | Gender, race, age, age squared, education level, marital status, home language, household size, female-headed household, mother’s education and household per capita income, have a child, in school |
| "Push" | Cape Area Panel Study | Zheng et al. (2018) | All: 2,644 Male: 1,197 Female: 1,477 | Low frequency: All: 26%  Male: 28% Female: 26%  High frequency All: 5% Male: 4% Female: 5% | Typical monthly wages (in South African Rands) | Not reported | Not reported | *Low frequency:* All: Coefficient (SE)=-0.14 (0.04), p<0.01 Male: Coefficient (SE)=-0.09 (0.05), p<0.1 Female: Coefficient (SE)=-0.19 (0.05), p<0.01  *High frequency:* All: Coefficient (SE)=-0.27 (0.06), p<0.01 Male: Coefficient (SE)=-0.19 (0.12), p>0.1 Female: Coefficient (SE)=-0.35 (0.06), p<0.01 | Gender, race, age, age squared, education level, marital status, home language, household size, female-headed household, mother’s education and household per capita income, have a child, in school |
|  |  |  |  |  | Employment | Not reported | Not reported | *Low frequency* All: Coefficient (SE)=-0.02 (0.06), p>0.1  *High frequency* All: Coefficient (SE)=-0.26 (0.13), p<0.05 | Gender, race, age, age squared, education level, marital status, home language, household size, female-headed household, mother’s education, and household per capita, income |
| "Hit hard" | Cape Area Panel Study | Zheng et al. (2018) | All: 2,644 Male: 1,197 Female: 1,477 | Low frequency: All: 10% Male: 11% Female: 10%  High frequency All: 2% Male: 2% Female: 2% | Typical monthly wages (in South African Rands) | Not reported | Not reported | Low frequency: All: Coefficient (SE)=-0.10 (0.05), p<0.1 Male: Coefficient (SE)=-0.09 (0.08), p>0.1 Female: Coefficient (SE)=-0.10 (0.07), p>0.1  High frequency: All: Coefficient (SE)=-0.22 (0.1), p<0.05 Male: Coefficient (SE)=-0.10 (0.16), p>0.1 Female: Coefficient (SE)=-0.29 (0.09), p<0.01 | Gender, race, age, age squared, education level, marital status, home language, household size, female-headed household, mother’s education and household per capita income, have a child, in school |
|  |  |  |  |  | Employment | Not reported | Not reported | *Low frequency* All: Coefficient (SE)=-0.02 (0.08), p>0.1  *High frequency* All: Coefficient (SE)=-0.22 (0.19), p>0.1 | Gender, race, age, age squared, education level, marital status, home language, household size, female-headed household, mother’s education, and household per capita, income |
| Corporal punishment | Zurich Project on the Social Development from Childhood into Adulthood (z-proso) | Averdijk et al. (2020) | 1,197 | Mean (SD)=1.15 (0.34) | Selling sexual services | Not reported | Not reported | OR=1.323, Unstandardized beta=0.280, p>=0.1 | Gender |
| ***Sexual violence*** | | | | | | | | | |
| Sexual abuse | Study of Alameda County Homeless Residents (STAR) Project | Tam et al. (2003) | 376 | 16.0% | Labour force participation | 50.0% | p>0.05 | Not reported | None |
| Sexual abuse | National Child Development Study (NCDS), the 1958 British birth cohort | Pereira et al. (2016) | 8,076 | 1.4% | Long-term sickness absence | Not reported | OR (95% CI)=3.51 (1.93-6.36)^§^ | OR (95% CI)=2.98 (1.62-5.47) | Sex, maternal age, birth weight, birth order, poor childhood health, social class in 1958, parental education, household amenities, crowding, and tenure, and other types of child maltreatment |
|  |  |  |  |  | NEET status | Not reported | OR (95% CI)=1.87 (1.07-3.25)^§^ | OR (95% CI)=1.70 (0.97-2.99) |  |
|  |  |  |  |  | Income-related support | Not reported | OR (95% CI)=2.15 (1.41-3.27)^§^ | OR (95% CI)=2.06 (1.35-3.14) |  |
|  |  |  |  |  | Manual occupation^¶^ | Not reported | OR (95% CI)=2.15 (1.37–3.36)^§^ | OR (95% CI)=1.73 (1.08–2.76) |  |
|  |  |  |  |  | Intergenerational social mobility (birth to 50 years) | Not reported | Not reported | Upward vs. stable manual: OR (95% CI)=0.50 (0.30-0.85) Downward vs. stable nonmanual: OR (95% CI)=1.85 (0.57-6.04) | Sex, other types of child maltreatment |
|  |  |  |  |  | Intragenerational social mobility (23 to 50 years) | Not reported | Not reported | Upward vs. stable manual: OR (95% CI)= 0.47 (0.22-1.01) Downward vs. stable nonmanual: OR (95% CI)=1.49 (0.71-3.13) |  |
| Sexual abuse | National Youth Survey Family Study (NYSFS) (formerly the National Youth Survey) | Macmillan (2000) | Not reported | Mean=0.16 (SD=0.37) | Income deficits in later adulthood | Not reported | Not reported | Unstandardised coefficient(SE) = -0.45 (0.33), p>0.05 | Gender, age at entry, family SES in adolescence, race, intact family, urban residence, suburban residence, delinquent peers, total delinquency |
| Sexual abuse | Christchurch Health and Development Study | Fergusson et al. (2013) | 984 | Non-contact sexual abuse: 2.8%  Contact sexual abuse: 5.3%  Sexual penetration: 6.5% | Welfare dependence | Non-contact sexual abuse: 21.4% Contact sexual abuse: 26.9% Sexual penetration: 37.5% | Test for trend: p<0.001 Beta (SE)=0.452 (0.084), p<0.001 | Beta (SE)=0.310 (0.099), p=0.002 Cohen's d=0.30, AR%=10.0 | Childhood physical punishment/maltreatment (<16 years); parental history of criminality, child IQ (8–9 years); number of changes of parents (0–16 years); maternal age at child’s birth |
|  |  |  |  |  | Income | Non-contact sexual abuse: 39.6 (27.6) Contact sexual abuse: 38.9 (23.9) Sexual penetration: 37.8 (30.2) | Test for trend: p<0.001  Beta (SE)=-4.605 (1.114), p<0.001 | Beta (SE)=-0.730 (1.102), p=0.508 Cohen's d=0.09 | Gender, family averaged standard of living (0-10 years), IQ (8-9 years), maternal age at child's birth |
| Sexual abuse | Christchurch Health and Development Study | Gibb et al. (2012) | 938-1009 | Non-contact abuse: 2.7% Contact sexual abuse: 5.1% Sexual penetration: 6.3% | Income | Not reported | Not reported | Not reported. Exposure not significant in the regression model for the effect of childhood family income on life course outcomes | N/A |
|  |  |  |  |  | Welfare dependence | Not reported | Not reported | Not reported. Exposure significant in the regression model for the effect of childhood family income on life course outcomes |  |
|  |  |  |  |  | Economic hardship | Not reported | Not reported | Not reported. Exposure significant in the regression model for the effect of childhood family income on life course outcomes |  |
|  |  |  |  |  | Working in paid employment | Not reported | Not reported | Not reported. Exposure not significant in the regression model for the effect of childhood family income on life course outcomes |  |
| Sexual abuse | TRacking Adolescents’ Individual Lives Survey (TRAILS) | de Vries et al. (2023) | 1,524 | Not reported | NEET | Not reported | Gamma (SE)=0.47 (0.13) | Not reported | N/A |
| Sexual abuse | Quebec Longitudinal Study of Kindergarten Children (QLSKC) | Bouchard et al. (2023) | 3,020 | 44.3% | Average employment earnings (USD) | No official report, missing self-report: $24,000 (19,100)  No official report, self-reported sexual abuse: $19,500 (15,500)  Official report, without/without self-report: $7,800 (11,500) | No official report, missing self-report: Beta (95% CI)=-3,181 (-5,236 to -1,127), p<0.05  No official report, self-reported sexual abuse: Beta (95% CI)=-9,107 (-12,274 to -5,940), p<0.05  Official report, without/without self-report: Beta (95% CI-26,993 (-38,929 to -15,055), p<0.05 | No official report, missing self-report: Beta (95% CI)=-1,906 (-3,912 to -98), p not less than 0.05  No official report, self-reported sexual abuse: Beta (95% CI)=-4,031 (-7,134 to -931), p<0.05  Official report, without/without self-report: Beta (95% CI)=-16,042 (-27,465 to -4,618), p<0.05 | Child’s sex, maternal and paternal education, parental earnings, parental mean age at childbirth, and family  unit (single- or 2-parent family) |
| Sexual abuse | Avon Longitudinal Study of Parents and Children (ALSPAC) | Smith et al. (2022) | 2,707 | 6.0% | Change in employment | Not reported | Ref. same/more hours:  Reduced hours: OR (95% CI)=1.27 (0.69-2.32)  Furlough/paid or unpaid leave: OR (95% CI)=1.61 (1.02-2.55)  Stopped working during pandemic: OR (95% CI)=0.97 (0.41-2.32)  Not working pre-pandemic: OR (95% CI)=1.23 (0.61-2.48) | Ref. same/more hours:  Reduced hours: OR (95% CI)=1.12 (0.6-2.10)  Furlough/paid or unpaid leave: OR (95% CI)=1.43 (0.89-2.32)  Stopped working during pandemic: OR (95% CI)=0.90 (0.36-2.22)  Not working pre-pandemic: OR (95% CI)=1.03 (0.49-2.17) | Ethnicity, age, home ownership, mother's marital status, parity, age at participant birth, and educational qualifications of mother and partner |
|  |  |  |  |  | Claimed benefits since pandemic | Not reported | OR (95% CI)=1.21 (0.70-2.08) | OR (95% CI)=1.17 (0.67 -2.05) | Ethnicity, age, home ownership, mother's marital status, parity, age at participant birth, and educational qualifications of mother and partner |
| Type of sexual abuse | Quebec Longitudinal Study of Kindergarten Children (QLSKC) | Bouchard et al. (2023) | All: 350  Male: 60  Female: 280  (numbers do not add up, not reported why) | Intrafamilial sexual abuse: 40.0% (not reported by sex) | Average employment earnings (USD) | Mean (SD)=$32,805 (20,153) | All: Beta (95% CI)=-6,898 (-11,823 to -1,973), p<0.05  Male: Beta (95% CI)=6,093 (-8,157 to 20,345), p>0.05  Female: Beta (95% CI)=-9,038 (-14,005 to -4,017), p<0.05 | All: Beta (95% CI)=-4,696 (-9,316 to -75), p<0.05  Male: Beta (95% CI)=7,466 (-5,540 to 20,472), p>0.05  Female: Beta (95% CI)=-7,184 (-11,943 to -2,423), p<0.05 | Child’s sex (when not stratified by sex), maternal and paternal education, parental earnings, parental mean age at childbirth, and family unit (single- or 2-parent family) |
| Severity of sexual abuse | Quebec Longitudinal Study of Kindergarten Children (QLSKC) | Bouchard et al. (2023) | All: 350  Male: 60  Female: 280  (numbers do not add up, not reported why) | Contact sexual abuse: 37.2% (not reported by sex) | Average employment earnings (USD) | Mean (SD)=$32,535 (18,713) | All: Beta (95% CI)=-5,603 (-11,934 to 726) p>0.05  Male: Beta (95% CI)=-2194 (-17,300 to 12,912), p>0.05  Female: Beta (95% CI)=-5926 (-12,857 to 1006), p>0.05 | All: Beta (95% CI)=-3,515 (-9,456 to 2,427), p>0.05  Male: Beta (95% CI)=-75 (-14,156 to 14,004), p>0.05  Female: Beta (95% CI)=-4400 (-10,967 to 2167), p>0.05 | Child’s sex (when not stratified by sex), maternal and paternal education, parental earnings, parental mean age at childbirth, and family unit (single- or 2-parent family) |
| Chronicity of sexual abuse | Quebec Longitudinal Study of Kindergarten Children (QLSKC) | Bouchard et al. (2023) | All: 350  Male: 60  Female: 280  (numbers do not add up, not reported why) | Multiple episodes: 17.1% (not reported by sex) | Average employment earnings (USD) | Mean (SD)=$32,130 (18,236) | All: Beta (95% CI)=-3,010 (-9,414 to 3,391) p>0.05  Male: Beta (95% CI)=22,558 (-7208 to 52,325)  Female: Beta (95% CI)=-3,508 (-9730 to 2713) | All: Beta (95% CI)=-2,081 (-8,056 to 3,892) p>0.05  Male: Beta (95% CI)=17,991 (-10,783 to 46,765)  Female: Beta (95% CI)=-3547 (-9418 to 2324) | Child’s sex (when not stratified by sex), maternal and paternal education, parental earnings, parental mean age at childbirth, and family unit (single- or 2-parent family) |
| ***Emotional violence*** | | | | | | | | | |
| Psychological abuse | National Child Development Study (NCDS), the 1958 British birth cohort | Pereira et al. (2016) | 8,076 | 9.6% | Long-term sickness absence | Not reported | OR (95% CI)=2.25 (1.67-3.03)^§^ | OR (95% CI)=2.20 (1.62-2.98) | Sex, maternal age, birth weight, birth order, poor childhood health, social class in 1958, parental education, household amenities, crowding, and tenure |
|  |  |  |  |  | NEET status | Not reported | OR (95% CI)=1.43 (1.10-1.85)^§^ | OR (95% CI)=1.41 (1.08-1.84) |  |
|  |  |  |  |  | Income-related support | Not reported | OR (95% CI)=1.41 (1.17-1.71)^§^ | OR (95% CI)=1.41 (1.16-1.71) |  |
|  |  |  |  |  | Manual occupation^¶^ | Not reported | OR (95% CI)=0.99 (0.83-1.19)^§^ | OR (95% CI)=0.92 (0.76-1.12) |  |
| Emotional abuse by parent/caregiver | Avon Longitudinal Study of Parents and Children (ALSPAC) | Cahill et al. (2022) | 14,964 | 5.7% | NEET | Not reported | Not reported | OR (95% CI)= 1.86 (0.74-4.67), p=0.179 | Sex, maternal age at birth, birthweight, gestation, maternal smoking in the 2nd trimester of pregnancy, parity, ethnicity of the child, socioeconomic status based on maternal and partner educational attainment, occupational social class, home ownership at birth, marital status at birth, mother BMI category pre-pregnancy, Alcohol Use Disorders Identification Test (AUDIT) measured when child was 17.5 years |
| Emotional abuse | Avon Longitudinal Study of Parents and Children (ALSPAC) | Smith et al. (2022) | 2,707 | 24.3% | Change in employment | Not reported | Ref. same/more hours:  Reduced hours: OR (95% CI)=1.03 (0.71-1.50)  Furlough/paid or unpaid leave: OR (95% CI)=1.29 (0.99-1.69)  Stopped working during pandemic: OR (95% CI)=1.13 (0.70-1.81)  Not working pre-pandemic: OR (95% CI)=1.19 (0.78-1.82) | Ref. same/more hours:  Reduced hours: OR (95% CI)=0.96 (0.65-1.42)  Furlough/paid or unpaid leave: OR (95% CI)=1.21 (0.91-1.60)  Stopped working during pandemic: OR (95% CI)=1.08 (0.66-1.76)  Not working pre-pandemic: OR (95% CI)=1.06 (0.68-1.66) | Ethnicity, age, home ownership, mother's marital status, parity, age at participant birth, and educational qualifications of mother and partner |
|  |  |  |  |  | Claimed benefits since pandemic | Not reported | OR (95% CI)=1.09(0.79-1.50) | OR (95% CI)=1.05 (0.75-1.47) | Ethnicity, age, home ownership, mother's marital status, parity, age at participant birth, and educational qualifications of mother and partner |
| Emotional abuse by parent | TRacking Adolescents’ Individual Lives Survey (TRAILS) | de Vries et al. (2023) | 1,524 | Not reported | NEET | Not reported | Gamma (SE)=0.29 (0.10) | Not reported | N/A |
| Emotional abuse ("put down" and/or "afraid of hurt") | Cape Area Panel Study | Zheng et al. (2018) | All: 2,644 Male: 1,197 Female: 1,477 | All: 54% Male: 52% Female: 55% | Typical monthly wages (in South African Rands) | Not reported | Not reported | All: Coefficient (SE)=-0.08 (0.04), p<0.05 Male: Coefficient (SE)=-0.03 (0.05), p>0.1 Female: Coefficient (SE)=-0.14 (0.06), p<0.05 | Gender, race, age, age squared, education level, marital status, home language, household size, female-headed household, mother’s education and household per capita income, have a child, in school |
| "Put down" | Cape Area Panel Study | Zheng et al. (2018) | All: 2,644 Male: 1,197 Female: 1,477 | Low frequency: All: 41% Male: 40% Female: 42%  High frequency: All: 8% Male: 7% Female: 9% | Typical monthly wages (in South African Rands) | Not reported | Not reported | *Low frequency:* All: Coefficient (SE)=-0.06 (0.04), p>0.1 Male: Coefficient (SE)=-0.03 (0.05), p>0.1 Female: Coefficient (SE)=-0.09 (0.05), p<0.1  *High frequency:* All: Coefficient (SE)=-0.16 (0.06), p<0.05 Male: Coefficient (SE)=-0.14 (0.08), p<0.1 Female: Coefficient (SE)=-0.17 (0.09), p<0.1 | Gender, race, age, age squared, education level, marital status, home language, household size, female-headed household, mother’s education and household per capita income, have a child, in school |
|  |  |  |  |  | Employment | Not reported | Not reported | *Low frequency:* All: Coefficient (SE)=0.01 (0.05), p>0.1  *High frequency:*  All: Coefficient (SE)=-0.06 (0.10), p>0.1 | Gender, race, age, age squared, education level, marital status, home language, household size, female-headed household, mother’s education, and household per capita, income |
| "Afraid of hurt" | Cape Area Panel Study | Zheng et al. (2018) | All: 2,644 Male: 1,197 Female: 1,477 | Low frequency: All: 25% Male: 24% Female: 26%  High frequency: All: 4% Male: 3% Female: 4% | Typical monthly wages (in South African Rands) | Not reported | Not reported | *Low frequency:* All: Coefficient (SE)=-0.08 (0.04), p<0.05 Male: Coefficient (SE)=-0.03 (0.05), p>0.1 Female: Coefficient (SE)=-0.09 (0.05), p<0.1  *High frequency:* All: Coefficient (SE)=-0.2 (0.08), p<0.05 Male: Coefficient (SE)=-0.30 (0.11), p<0.01 Female: Coefficient (SE)=-0.14 (0.11), p>0.1 | Gender, race, age, age squared, education level, marital status, home language, household size, female-headed household, mother’s education and household per capita income, have a child, in school |
|  |  |  |  |  | Employment | Not reported | Not reported | Low frequency: All: Coefficient (SE)=-0.03 (0.06), p>0.1  *High frequency:*  All: Coefficient (SE)=-0.07 (0.13), p>0.1 | Gender, race, age, age squared, education level, marital status, home language, household size, female-headed household, mother’s education, and household per capita, income |
| ***Neglect*** | | | | | | | | | |
| Neglect | National Child Development Study (NCDS), the 1958 British birth cohort | Pereira et al. (2016) | 8,076 | 16.0% | Long-term sickness absence | Not reported | OR (95% CI)=2.50 (1.94-3.22)^§^ | OR (95% CI)=1.77 (1.34-2.33) | Sex, maternal age, birth weight, birth order, poor childhood health, social class in 1958, parental education, household amenities, crowding, and tenure |
|  |  |  |  |  | NEET status | Not reported | OR (95% CI)=1.67 (1.32-2.13)^§^ | OR (95% CI)=1.47 (1.13-1.90) |  |
|  |  |  |  |  | Income-related support | Not reported | OR (95% CI)=1.17 (0.97-1.40)^§^ | OR (95% CI)=1.06 (0.88-1.28) |  |
|  |  |  |  |  | Manual occupation | Not reported | OR (95% CI)=2.54 (2.22-2.91)^§^ | OR (95% CI)=1.78 (1.53-2.06) |  |
|  |  |  |  |  | Intergenerational social mobility (birth to 50 years) | Not reported | Not reported | Upward vs. stable manual: OR (95% CI)=0.45 (0.39-0.53) Downward vs. stable nonmanual: OR (95% CI)=2.31 (1.56-3.41) | Sex, other types of child maltreatment |
|  |  |  |  |  | Intragenerational social mobility (23 to 50 years) | Not reported | Not reported | Upward vs. stable manual: OR (95% CI)=0.61 (0.50-0.74) Downward vs. stable nonmanual: OR (95% CI)=2.11 (1.63-2.74) |  |
| Neglect | National Child Development Study (NCDS), the 1958 British birth cohort | Fahy et al. (2017) | 9,137 | 9.8% | Labour participation: part-time work | Not reported | Not reported | RR (95% CI)=0.85 (0.60-1.20), p≥0.05 | Gender and childhood mental health |
|  |  |  |  |  | Labour participation: unemployed | Not reported | Not reported | RR (95% CI)=1.46 (0.85-2.53), p≥0.05 |  |
|  |  |  |  |  | Labour participation: permanent sick | Not reported | Not reported | RR (95% CI)=2.96 (1.99-4.41), p<0.05 |  |
|  |  |  |  |  | Labour participation: retired | Not reported | Not reported | RR (95% CI)=0.31 (0.11-0.91), p<0.05 |  |
|  |  |  |  |  | Labour participation: homemaker/other | Not reported | Not reported | RR (95% CI)=1.18 (0.77-1.81), p≥0.05 |  |
| Physical neglect | National Child Development Study (NCDS), the 1958 British birth cohort | Schurer et al. (2017) | 4132 | Not reported | Net individual earnings | Not reported | Beta (SE)=−0.274 (0.086), p<0.01 | Beta (SE)-0.219 (0.084), p<0.01 | Female, low birth weight, premature birth, mother’s age at birth, birth order, father’s social class, father’s and mother’s age when he/she left full-time education, father’s income bands (missing for 42% of cohort members) |
|  |  |  | 7450 | Not reported | Welfare dependence | Not reported | Beta (SE)=0.137 (0.021), p<0.01 | Beta (SE)=0.118 (0.021), p<0.01 |  |
| Emotional neglect | National Child Development Study (NCDS), the 1958 British birth cohort | Pereira et al. (2016) | 8,076 | 10.9% | Long-term sickness absence | Not reported | OR (95% CI)=1.76 (1.30-2.39)^§^ | OR (95% CI)=1.60 (1.18-2.18) | Sex, maternal age, birth weight, birth order, poor childhood health, social class in 1958, parental education, household amenities, crowding, and tenure |
|  |  |  |  |  | NEET status | Not reported | OR (95% CI)=1.41 (1.09-1.82)^§^ | OR (95% CI)=1.35 (1.05-1.75) |  |
|  |  |  |  |  | Income-related support | Not reported | OR (95% CI)=1.19 (0.98-1.44)^§^ | OR (95% CI)=1.16 (0.95-1.40) |  |
|  |  |  |  |  | Manual occupation^¶^ | Not reported | OR (95% CI)=1.26 (1.07-1.48)^§^ | OR (95% CI)=1.14 (0.96-1.35) |  |
|  |  |  |  |  | Intergenerational social mobility (birth to 50 years) | Not reported | Not reported | Upward vs. stable manual: OR (95% CI)=0.87 (0.71-1.06) Downward vs. stable nonmanual: OR (95% CI)=1.15 (0.77-1.72) | Sex, other types of child maltreatment |
|  |  |  |  |  | Intragenerational social mobility (23 to 50 years) | Not reported | Not reported | Upward vs. stable manual: OR (95% CI)=0.96 (0.75-1.24) Downward vs. stable nonmanual: OR (95% CI)=1.16 (0.85-1.58) |  |
| Emotional neglect | Taiwan Youth Project Surveys | Lin and Chiao (2021) | All: 2,897 Male: 1,433 Female: 1,464 | Not reported | NEET | Not reported | Not reported | All: OR (SE)=1.20 (0.35), p not less than 0.05 Males: OR (SE)=1.14 (0.45), p not less than 0.1 Female: OR (SE)=1.30 (0.53), p not less than 0.1 | Sex, school year at enrolment, county, education level, only child, individual covariates (early pubertal maturation, lack of sleep, substance use, educational aspiration, class rank, depressive symptoms, self-esteem), family covariates (parental control, family cohesion) |
| Emotional neglect | Avon Longitudinal Study of Parents and Children (ALSPAC) | Cahill et al. (2022) | 14,964 | 15.2% | NEET | Not reported | Not reported | OR (95% CI)=1.26 (0.83-1.93), p=0.270 | Sex, maternal age at birth, birthweight, gestation, maternal smoking in the 2nd trimester of pregnancy, parity, ethnicity of the child, socioeconomic status based on maternal and partner educational attainment, occupational social class, home ownership at birth, marital status at birth, mother BMI category pre-pregnancy, Alcohol Use Disorders Identification Test (AUDIT) measured when child was 17.5 years |
| Emotional neglect | Avon Longitudinal Study of Parents and Children (ALSPAC) | Smith et al. (2022) | 2,707 | 19.5% | Change in employment | Not reported | Ref. same/more hours:  Reduced hours: OR (95% CI)=1.41 (0.98-2.03)  Furlough/paid or unpaid leave: OR (95% CI)=1.44 (1.08-1.92)  Stopped working during pandemic: OR (95% CI)=0.95(0.55-1.63)  Not working pre-pandemic: OR (95% CI)=1.75 (1.16-2.66) | Ref. same/more hours:  Reduced hours: OR (95% CI)=1.36 (0.93-1.99)  Furlough/paid or unpaid leave: OR (95% CI)=1.34 (0.99-1.8)  Stopped working during pandemic: OR (95% CI)=0.98 (0.56-1.73)  Not working pre-pandemic: OR (95% CI)=1.67 (1.08-2.57) | Ethnicity, age, home ownership, mother's marital status, parity, age at participant birth, and educational qualifications of mother and partner |
|  |  |  |  |  | Claimed benefits since pandemic | Not reported | OR (95% CI)=0.95 (0.67-1.35) | OR (95% CI)=0.94 (0.66-1.35) | Ethnicity, age, home ownership, mother's marital status, parity, age at participant birth, and educational qualifications of mother and partner |
| ***Bullying*** | | | | | | | | | |
| Bullying involvement | Youth and Mental Health Study | Sigurdson et al. (2014) | 1,266 | Being bullied: 4.6% Bully victims (bullied others and been bullied): 3.1% | Occupational status: Disability/social help/unemployed | Being bullied: 9.7% Bully victims: 11.4% | Being bullied: OR (95% CI)=1.72 (0.94-3.14), p>0.05 Bully victims: OR (95% CI)=1.81 (0.61-5.38), p>0.05 | Being bullied: OR (95% CI)=1.74 (0.94-3.22), p>0.05 Bully victims: OR (95% CI)=2.5 (0.83-7.72), p>0.05 | Gender, parental SES |
| Bullying involvement | The Great Smoky Mountain Study | Wolke et al. (2013) | 1,273 | Being bullied: 23.6% Bully victims: 6.1% | Poverty | Being bullied: 29.9% Bully victims: 38.2% | Being bullied: OR (95% CI)=1.91 (1.15-3.17), p=0.013 Bully victims: OR (95% CI)=2.76 (1.17-6.55), p=0.021 | Not reported | N/A |
|  |  |  |  |  | Dismissal from a job | Being bullied: 33.5% Bully victims: 38.1% | Being bullied: OR (95% CI)=1.99 (1.23, 3.21), p=0.005 Bully victims: OR (95% CI)=2.43 (1.06-5.61), p=0.037 | Not reported |  |
|  |  |  |  |  | Quitting multiple jobs | Being bullied: 20.3% Bully victims: 37.3% | Being bullied: OR (95% CI)=2.33 (1.28, 4.26), p=0.006 Bully victims: OR (95% CI)=5.44 (2.28-12.96), p<0.001 | Not reported |  |
| Being bullied | National Child Development Study (NCDS), the 1958 British birth cohort | Brimblecombe et al. (2018) | All: 8,581 Male: 4,434 Female: 4,147 | Occasionally bullied (female): 26.2% Occasionally bullied (male): 28.5% Frequently bullied (female): 12.3% Frequently bullied (male): 15.8% | Unemployed or economically inactive | Occasionally bullied (female): 8.7% Occasionally bullied (male): 9.2% Frequently bullied (female): 11.9% Frequently bullied (male): 14.0% | OR (95% CI)=1.44 (1.09-1.91), p<0.05 OR (95% CI)=1.21 (0.92-1.58), p≥0.1 OR (95% CI)=1.85 (1.32-2.58), p<0.05 OR (95% CI)=2.06 (1.56-2.72), p<0.05 | OR (95% CI)=1.34 (0.97-1.84), p<0.1 OR (95% CI)=0.90 (0.64-1.27), p≥0.1 OR (95% CI)=1.39 (0.94-2.06), p<0.1 OR (95% CI)=1.49 (1.04-2.13), p<0.05 | Family social class, adversity, low parental involvement, childhood IQ, childhood emotional and behavioural problems |
|  |  |  | All: 7,323 Male: 2,919 Female: 3,284 (Numbers do not add up in paper) |  | Earnings | Occasionally bullied (female): £281.27 Occasionally bullied (male): £524.38 Frequently bullied (female): £258.50 Frequently bullied (male): £464.21 | Mean difference=−21.05* (−37.76, −4.34), p<0.05 Mean difference=−23.49 (-68.27, 21.28), p≥0.1 Mean difference=−41.92 (−60.89, −22.96), p<0.05 Mean difference=−70.34 (−105.90, −34.80), p<0.05 | Mean difference=−8.72 (−25.92, 8.48), p≥0.1 Mean difference=−7.72 (−49.10, 34.66), p≥0.1 Mean difference=−22.74 (−42.05, −3.43), p<0.1 Mean difference=−8.55 (−46.98, 29.88), p≥0.1 |  |
| Being bullied | National Child Development Study (NCDS), the 1958 British birth cohort | Brown (2008) | 3,971 | Bullied at age 7: 34.5% Bullied at age 11: 23.8% | Log wages | Mean at age 7: Never bullied: 5.926  Sometimes bullied: 5.903 Frequently bullied: 5.841  Mean at age 11: Never bullied: 5.916  Sometimes bullied: 5.926 Frequently bullied: 5.823 | Not reported | Age 7: Beta= -0.0305 (Tstat: 3.23) Age 11: Beta= -0.0283 (Tstat: 2.77) | Gender, a quadratic in labour market experience, marital status, industry, occupation, firm size, part-time employment, highest educational attainment |
| Being bullied | National Child Development Study (NCDS), the 1958 British birth cohort | Takizawa et al. (2014) | 7,771 | 27.4% | Current unemployment | Males: 3.5% Females: 2.3% | Not reported | Males: Beta (95% CI)=0.98 (0.61-1.57) Females: Beta (95% CI)=0.94 (0.51-1.73) | Childhood confounders (childhood IQ, internalizing and externalizing problems, family social class, childhood adversities [poverty; parental mental health, drug, or alcohol problems; family conflict; physical and sexual abuse]), ever in care of local authority or voluntary agency, parental involvement) |
|  |  |  |  |  | Weekly net pay | Males: 317.4 (340.4) Females: 195.7 (202.7) | Not reported | Males: Beta (95% CI)=-1.13 (-27.1 to 24.9) Females: Beta (95% CI)=4.88 (-10.2 to 19.9) |  |
| Being bullied | National Child Development Study (NCDS), the 1958 British birth cohort | Blanchflower et al. (2024) | 23 years: 10,960  33 years: 9,971  42 years: 9,992  46 years: 8,390  50 years: 8,571  55 years: 7,738 | Of individuals responding to bullying question at age 11:  Bullied sometimes: 21%  Bullied frequently: 4% | Current employment (self-report) | Not reported | Not reported | Bullied sometimes:  23 years: Coefficient (t-statistic)=-0.0193 (1.86)  33 years: Coefficient (t-statistic)=-0.390 (3.74)  42 years: Coefficient (t-statistic)=-0.183 (1.92)  46 years: Coefficient (t-statistic)=-0.0159 (1.63)  50 years: Coefficient (t-statistic)=-0.125 (1.19)  55 years: Coefficient (t-statistic)=-0.0186 (1.54)  Bullied frequently:  23 years: Coefficient (t-statistic)=-0.0393 (1.75)  33 years: Coefficient (t-statistic)=-0.0478 (2.14)  42 years: Coefficient (t-statistic)=-0.0110 (0.54)  46 years: Coefficient (t-statistic)=-0.0229 (1.06)  50 years: Coefficient (t-statistic)=-0.0692 (3.05)  55 years: Coefficient (t-statistic)=-0.0634 (2.44) | Sex, Bristol Social Adjustment Guide (BSAG) total score at 7, IQ score at 7 |
| Being bullied | Avon Longitudinal Study of Parents and Children (ALSPAC) | Cahill et al. (2022) | 14,964 | 15.5% | NEET | Not reported | Not reported | OR=1.43 (1.05-1.96), p=0.025 | Sex, maternal age at birth, birthweight, gestation, maternal smoking in the 2nd trimester of pregnancy, parity, ethnicity of the child, socioeconomic status based on maternal and partner educational attainment, occupational social class, home ownership at birth, marital status at birth, mother BMI category pre-pregnancy, Alcohol Use Disorders Identification Test (AUDIT) measured when child was 17.5 years |
| Being bullied | Avon Longitudinal Study of Parents and Children (ALSPAC) | Smith et al. (2022) | 2,707 | 23.9% | Change in employment | Not reported | Ref. same/more hours:  Reduced hours: OR (95% CI)=1.15 (0.79-1.67)  Furlough/paid or unpaid leave: OR (95% CI)=1.29 (0.99-1.68)  Stopped working during pandemic: OR (95% CI)=1.19 (0.79-1.81)  Not working pre-pandemic: OR (95% CI)=1.31 (0.90-1.92) | Ref. same/more hours:  Reduced hours: OR (95% CI)=1.11 (0.76-1.63)  Furlough/paid or unpaid leave: OR (95% CI)=1.24 (0.94-1.62)  Stopped working during pandemic: OR (95% CI)=1.21 (0.79-1.87)  Not working pre-pandemic: OR (95% CI)=1.19 (0.79-1.8) | Ethnicity, age, home ownership, mother's marital status, parity, age at participant birth, and educational qualifications of mother and partner |
|  |  |  |  |  | Claimed benefits since pandemic |  |  |  |  |
| Being bullied | Next Steps | Tayfur et al. (2022) | 2,125 | Not reported | NEET | Not reported | OR=0.54 (0.36, 0.79), p=0.002 | OR=0.57 (0.36, 0.90), p=0.017 | Sex, ethnicity, socioeconomic status, caring responsibility, and family composition |
| Being bullied (binary) | Next Steps | Gorman et al. (2021) | All: 7,569 | Not reported | Weekly income | Bullied once: Mean=5.67;  Repeatedly bullied: Mean=5.72 | Not reported | All: Beta (SE)=0.023 (0.004), p<0.01 Beta (SE)=0.010 (0.003), p<0.01* | Gender, ethnicity, birth month, Government Office Region and English being a second language, school fixed effects *Additionally adjusted for controls at age 14: local area deprivation, parental information including age, education, health, income and marital status, test scores at age 10 (KS2) and whether the school was the parent’s first choice school |
|  |  |  | Males: 3,570 | Not reported | Weekly income | Not reported | Not reported | Males: Beta (SE)=-0.00710 (0.00478), p not less than 0.01 | Gender, ethnicity, birth month, Government Office Region and English being a second language, school fixed effects, controls at age 14: local area deprivation, parental information including age, education, health, income and marital status, test scores at age 10 (KS2) and whether the school was the parent’s first choice school |
|  |  |  | Females: 2,843 | Not reported | Weekly income | Not reported | Not reported | Females: Beta (SE)=-0.0108 (0.00592), p<0.10 |  |
|  |  |  | All: 7,569 | Not reported | Unemployment | Bullied once: Mean=0.11 Repeatedly bullied: Mean=0.12 | Not reported | All: Beta (SE)=0.035 (0.007), p<0.01 Beta (SE)=0.028 (0.007), p<0.01* | Gender, ethnicity, birth month, Government Office Region and English being a second language, school fixed effects *Additionally adjusted for controls at age 14: local area deprivation, parental information including age, education, health, income and marital status, test scores at age 10 (KS2) and whether the school was the parent’s first choice school |
|  |  |  | Males: 3,570 | Not reported | Unemployment | Not reported | Not reported | Males: Beta (SE)=0.0210 (0.0118), p<0.10 | Gender, ethnicity, birth month, Government Office Region and English being a second language, school fixed effects, controls at age 14: local area deprivation, parental information including age, education, health, income and marital status, test scores at age 10 (KS2) and whether the school was the parent’s first choice school |
|  |  |  | Females: 2,843 | Not reported | Unemployment | Not reported | Not reported | Females: Beta (SE)=0.0383 (0.00972), p<0.01 |  |
| Being bullied/bullying factor (continuous) | Next Steps | Gorman et al. (2021) | All: 7,569 | Not reported | Weekly income | Not reported | Not reported | All: Beta (SE)=-0.018 (0.003), p<0.01 Beta (SE)=-0.007 (0.003), p<0.05* | Gender, ethnicity, birth month, Government Office Region (GOR) and English being a second language, school fixed effects *Additionally adjusted for controls at age 14: local area deprivation, parental information including age, education, health, income and marital status, test scores at age 10 (KS2) and whether the school was the parent’s first choice school |
|  |  |  | Males: 3,570 | Not reported | Weekly income | Not reported | Not reported | Males: Beta (SE)=-0.00556 (0.00419), p not less than 0.1 | Gender, ethnicity, birth month, Government Office Region (GOR) and English being a second language, school fixed effects, controls at age 14: local area deprivation, parental information including age, education, health, income and marital status, test scores at age 10 (KS2) and whether the school was the parent’s first choice school |
|  |  |  | Females: 2,843 | Not reported | Weekly income | Not reported | Not reported | Females: Beta (SE)=-0.00549 (0.00351), p not less than 0.1 |  |
|  |  |  | All: 7,569 | Not reported | Unemployment | Not reported | Not reported | All: Beta (SE)=0.020 (0.006), p<0.01 Beta (SE)=0.011 (0.006), p<0.1* | Gender, ethnicity, birth month, Government Office Region (GOR) and English being a second language, school fixed effects *Additionally adjusted for controls at age 14: local area deprivation, parental information including age, education, health, income and marital status, test scores at age 10 (KS2) and whether the school was the parent’s first choice school |
|  |  |  | Males: 3,570 | Not reported | Unemployment | Not reported | Not reported | Males: Beta (SE)=0.0103 (0.0115), p not less than 0.1 | Gender, ethnicity, birth month, Government Office Region (GOR) and English being a second language, school fixed effects, controls at age 14: local area deprivation, parental information including age, education, health, income and marital status, test scores at age 10 (KS2) and whether the school was the parent’s first choice school |
|  |  |  | Females: 2,843 | Not reported | Unemployment | Not reported | Not reported | Females: Beta (SE)=0.00912 (0.00632), p not less than 0.1 |  |
| Bullied under 12 | National Longitudinal Survey of Youth 1997 (NLSY) | Mukerjee (2018) | 2,006 | Not reported | Income | Female: Mean=$21,510.35 Male: Mean=$31,433.70 | Not reported | b (SE)=0.0329 (0.0435), p>0.1 b (SE)=0.0579 (0.0422), p>0.1* | Education, race, ethnicity, age, height, health, labour market indicators, marital status variables, SES during childhood, personal characteristics or problems that maybe related to bullying, whether still enrolled in school, and geographical regions *Additionally adjusted for occupation |
| Bullied teen | National Longitudinal Survey of Youth 1997 (NLSY) | Mukerjee (2018) | 2,006 | Not reported | Income | Female: Mean=$27,483.95 Male: Mean=23,886,62 | Not reported | b (SE)=−0.1810 (0.0645), p<0.01 b (SE)=-0.2226 (0.0876); p<0.01* | Education, race, ethnicity, age, height, health, labour market indicators, marital status variables, SES during childhood, personal characteristics or problems that maybe related to bullying, whether still enrolled in school, and geographical regions *Additionally adjusted for occupation |
| Experience of aggressive behaviour in school | Next Steps | Karyda (2020) | 5,660 | 41.9% | NEET | 24.0% | Not reported | OR=1.288 | Area characteristics (crime in the neighbourhood), sex, ethnicity, family background indicators, priori educational attainment, engagement in risky behaviour, police involvement |
| ***Adolescent IPV*** | | | | | | | | | |
| Adolescent IPV | National Longitudinal Study of Adolescent to Adult Health (ADD Health) | Heng (2015) | 1,386 | 32.7% | Employment status | Not reported | Spearman correlation=-0.05, p>0.05 | Not reported | N/A |
| Adolescent IPV | Women’s Employment Study | Adams et al. (2013) | 498 | 16.0% | Earnings | Not reported | T1: Correlation=-0.61, p>0.05 T2: Correlation=-0.62, p>0.05 T3: Correlation=-0.095, p<0.05 | Not reported | N/A |
| Violence between child and partner | Avon Longitudinal Study of Parents and Children (ALSPAC) | Cahill et al. (2022) | 14,964 | 14.2% | NEET | Not reported | Not reported | OR (95%CI)=1.69 (0.97-2.92), p=0.062 | Sex, maternal age at birth, birthweight, gestation, maternal smoking in the 2nd trimester of pregnancy, parity, ethnicity of the child, socioeconomic status based on maternal and partner educational attainment, occupational social class, home ownership at birth, marital status at birth, mother BMI category pre-pregnancy, Alcohol Use Disorders Identification Test (AUDIT) measured when child was 17.5 years |
| ***Witnessing violence*** | | | | | | | | | |
| Witnessing parental violence | National Youth Survey Family Study (NYSFS) | Covey et al. (2013) | Unclear: 1,266 for Wave 10 and 1173 for Wave 11 | Inconsistent reporting: 10.3% Consistent reporting: 15.1% | Employment | Not reported | Inconsistent reporting: r=-0.009, p>0.05 Consistent reporting: r=-0.062, p>0.06 | Inconsistent reporting: Beta (SE)=-0.221 (0.355), p=0.532; OR=0.801 Consistent reporting: Beta (SE)=-0.518 (0.308), p=0.093 OR=0.596 | Sex, race/ethnicity, place of residence, family structure, Hollingshead index of social position |
|  |  |  |  |  | Income | Not reported | Inconsistent reporting: r=-0.028, p>0.05 Consistent reporting: r=-0.99, p<0.05 | Inconsistent reporting: Beta (SE)=-0.061 (0.168), p=0.716 Consistent reporting: Beta (SE)=-0.312 (0.159), p=0.05 |  |
| Parental DVA (child not aware) | Avon Longitudinal Study of Parents and Children (ALSPAC) | Herbert et al. (2023) | 6,252 | Male: 7.0%  Female: 7.6% | Mean earnings loss | Mean earnings=£1,578.18 | Not reported | Coefficient=-6.69 (97.11, -110.5), p>0.05 | Ethnicity, sexual orientation, deprivation subject to IPV as young person, maternal qualifications |
| Parental DVA (child aware) | Avon Longitudinal Study of Parents and Children (ALSPAC) | Herbert et al. (2023) | 6,252 | Male: 3.0%  Female: 3.9% | Mean earnings loss | Mean earnings=£1,577.65 | Not reported | Coefficient=95.69 (346.84, -155.46), p>0.05 | Ethnicity, sexual orientation, deprivation subject to IPV as young person, maternal qualifications |
| Witnessing IPV | National Child Development Study (NCDS), the 1958 British birth cohort | Pereira et al. (2016) | 8,076 | 5.7% | Long-term sickness absence | Not reported | OR (95% CI)=1.97 (1.34-2.89)^§^ | OR (95% CI)=1.62 (1.09-2.40) | Sex, maternal age, birth weight, birth order, poor childhood health, social class in 1958, parental education, household amenities, crowding, and tenure |
|  |  |  |  |  | NEET status | Not reported | OR (95% CI)=1.74 (1.28-2.35)^§^ | OR (95% CI)=1.59 (1.17-2.16) |  |
|  |  |  |  |  | Income-related support | Not reported | OR (95% CI)=1.55 (1.22-1.97)^§^ | OR (95% CI)=1.47 (1.16-1.88) |  |
|  |  |  |  |  | Manual occupation^¶^ | Not reported | OR (95% CI)=1.32 (1.05-1.66)^§^ | OR (95% CI)=1.03 (0.81-1.31) |  |
| Witnessing a very violence event | N/A | Lund et al. (2013) | 3,058 | 9.7% | Labour market participation | 25.6% | Female: OR (95% CI)=2.21 (1.46-3.33) Male: OR (95% CI)=1.51 (1.02-2.21) | Female: OR (95% CI)=2.23 (1.47-3.37) Male: OR (95% CI)=1.52 (1.03-2.23) Female: OR (95% CI)=2.56 (1.61-4.08)* Male: OR (95% CI)=1.53 (0.98-2.40)* Female: OR (95% CI)=2.47 (1.54-3.94)† Male: OR (95% CI)=1.51 (0.95-2.38)† | Socio-economic status |
| ***Composite measures*** | | | | | | | | | |
| Childhood victimisation | National Longitudinal Survey of Youth 1997 (NLSY) | Fernandez et al. (2015) | 8,901 | 50.5% Mean (SE)=0.91 (0.01), 95% CI=0.88-0.94 | Occupational prestige ("pooled time") | Not reported | Not reported | Female: Beta (SE)=0.09 (0.09), p=0.305 Male: Beta=0.53; p<0.01 (SE Not reported) | Sex, age, race, ethnicity, number of years participants had two biological parents in the house, highest educational grade attained by the participant and their parents, school enrolment status |
|  |  |  |  |  | Annual income ("pooled time") | Not reported | Not reported | Beta (SE)=-$301 ($154), p=0.05 |  |
|  |  |  |  |  | Occupational prestige at time point | Not reported | Not reported | Beta (SE)=0.65 (0.13), p<0.001 |  |
|  |  |  |  |  | Annual income at time point | Not reported | Not reported | Beta (SE)=$1,003 ($175), p<0.001 |  |
|  |  |  |  |  | Trajectory of occupational prestige | Not reported | Not reported | Beta (SE)=-0.10 (0.02), p<0.001 |  |
|  |  |  |  |  | Trajectory of annual income | Not reported | Not reported | Beta (SE)=-$259 ($33), p<0.001 |  |
| Adolescent victimisation | National Youth Survey Family Study (NYSFS) | Macmillan (1998) | 1,725 | 37.5% | Occupational status | 31.7% | Not reported | b (SE)=-2.914 (1.175), p<0.01 b (SE)=-0.878 (1.217), p>0.05* | Age, sex, family SES, black, urban residence, suburban residence, neighbourhood quality, residential stability, number of children, intact family, familial control, family-peer integration, family support, delinquency *Additionally adjusted for educational effort, educational investment, grade point average, and educational attainment |
|  |  |  |  |  | Labour force participation | 88.3% | Not reported | b (SE)=-0.735 (0.218), p<0.01 b (SE)=-0.741 (0.253), p<0.01* |  |
|  |  |  |  |  | Unemployed | 2.9% | Not reported | b (SE)=0.897 (0.416), p<0.01 b (SE)=0.955 (0.4980, p<0.05* |  |
|  |  |  |  |  | On public assistance | 4.3% | Not reported | b (SE)=1.095 (0.355), p<0.01 b (SE)0.777 (0.432), p<0.05* |  |
|  |  |  |  |  | Average hourly wage | 6.6% | Not reported | b (SE)=-0.593 (0.248), p<0.01 b (SE)=-0.388 (0.274), p>0.05* |  |
| Adolescent victimisation | National Youth Survey Family Study (NYSFS) | Macmillan and Hagan (2004) | Not reported | 50% (almost 15% reported one victimization incident, 15% reported two or three incidents, and 20% reported four or more incidents) | Occupational status | Not reported | Not reported | b (SE)=-6.673 (1.289); beta=0.153, p<0.001 b (SE)=-2.686 (1.334); beta=-0.059, p<0.01* b (SE)=-0.010 (1.315); beta=0.000, p>0.05† | Delinquency *Additionally adjusted for age, sex, family SES, black, urban residence, suburban residence, neighborhood quality, residential stability, number of children, intact family, familial control, family-peer integration, family support †Additionally adjusted for sociodemographic variables above, plus educational commitment, educational effort, grade point average, education attainment |
|  |  |  |  |  | Labour force participation | Not reported | Not reported | Beta (SE)=-0.309 (0.187); OR=0.735, p>0.05 Beta (SE)=-0.710 (0.223); OR=0.492, p<0.001* Beta (SE)=0.569 (0.250); OR=0.566, p<0.01† |  |
|  |  |  |  |  | Unemployment | Not reported | Not reported | Beta (SE)=0.445 (0.306); OR=1.56, p>0.05 Beta (SE)=0.842 (0.368); OR=2.32, p<0.01* Beta (SE)=0.833 (0.396); OR=2.300, p<0.01† |  |
|  |  |  |  |  | On public assistance | Not reported | Not reported | Beta (SE)=0.501 (0.256); OR=2.158, p<0.01 Beta (SE)=0.769 (0.345); OR=2.158, p<0.01* Beta (SE)=0.317(0.421); OR=1.373, p>0.05† |  |
|  |  |  |  |  | Average hourly wage | Not reported | Not reported | b (SE)=-0.091 (0.028); beta=-0.098, p<0.001  b (SE)=-0.059 (0.029); beta=-0.062, p<0.001* b (SE)=-0.014 (0.029), beta=-0.14, p>0.05† |  |
| Adolescent victimisation | National Youth Survey Family Study (NYSFS) | Macmillan (2000) | Not reported | Mean=0.38 (SD=0.48) | Income deficits in later adulthood | Not reported | Not reported | Unstandardised coefficient(SE) = -1.03 (0.27), p<0.001 | Gender, age at entry, family SES in adolescence, race, intact family, urban residence, suburban residence, deliquent peers, total delinquency |
| Adolescent victimisation | Toronto Urban Youth Project | Macmillan (1998) | 566 | 14.0% | Occupational status | Low occupational status: 48.5% Medium occupational status: 33.3%  High occupational status: 18.2% | Not reported | b (SE)=-4.784 (1.493), p<0.01 b (SE)=-2.866 (1.506), p>0.05* | Age, sex, father's occupational status, mother's occupational status, ability, working mother, number of siblings, family structure, father's participation, mother's support, father's support, father's control, mother's control, substructure of delinquency, party subculture *Additionally adjusted for educational investment, educational performance (grades), educational aspirations, and overall educational attainment |
| Violent victimisation | Zurich Project on the Social Development from Childhood into Adulthood (z-proso) | Averdijk et al. (2020) | 1,197 | Mean (SD)= 0.59 (0.87) | Selling sexual services | Not reported | Not reported | OR=1.759, Unstandardized beta=0.565, p<0.01 | Gender |
| Peer victimisation | Not reported | Day et al. (2017) | 89 | Mean (SD)=4.49 (2.02) | Employed | Not reported | OR (95% CI)=0.92 (0.63-1.33) | OR (95% CI)=0.87 (0.59-1.27) | Participant sex, familial SES, family functioning, and childhood emotional and behavioural problems |
| Child maltreatment | National Child Development Study (NCDS), the 1958 British birth cohort | Conti et al. (2021) | 7,043 (models 1-3) 7,042 (model 4) | 20.6% | Employed (50 years) | Not reported | Not reported | Beta (SE)=-0.056 (0.011), p<0.01 Beta (SE)=-0.047 (0.011), p<0.01* Beta (SE)=-0.043 (0.011), p<0.01† Beta (SE)=-0.036 (0.012), p<0.01‡ | Gender, ethnicity, interview date (year, month, day) *Additionally adjusted for variables recorded at birth: the social class of the husband and of the father of the mother, parity, birthweight, whether the mother stayed in school beyond the minimum school leaving age, marital status, smoking in pregnancy, mother’s and father’s age at the child’s birth, working during pregnancy, weight before pregnancy, mother’s height, whether the mother had any antenatal visit in the first trimester of pregnancy, and the number of people per room. †Additional adjusted for other early adverse childhood experiences collected at 42 years: whether the child lived with both natural parents, whether there was any family contacts with the probation officer, whether the parents divorced, whether a parent was alcoholic, whether the death of the father or of the mother occurred, whether there was any domestic tension, whether there were financial or housing difficulties, and the number of times the family moved since the birth of the child.  ‡Additionally adjusted for extended set of early adverse childhood experiences, reported retrospectively in the biomedical sweep: whether the mother suffered from nervous or emotional trouble, whether the mother had trouble drinking, whether there was some or a lot of conflict and tension in the house, whether the child grew up in poverty or in financial hardship. |
|  |  |  | 6,604 (models 1-3) 6,603 (model 4) | 20.6% | Employed (55 years) | Not reported | Not reported | Beta (SE)=-0.041 (0.013), p<0.01 Beta (SE)=-0.034 (0.013), p<0.01* Beta (SE)=-0.031 (0.013), p<0.05† Beta (SE)=-0.009 (0.014), p not less than 0.1‡ |  |
|  |  |  | 4,997 (models 1-3) 4,996 (model 4) | 20.6% | Net weekly earnings if employed (50 years) | Not reported | Not reported | Beta (SE)=-9.98 (10.96), p not less than 0.1 Beta (SE)=-2.60 (10.91), p not less than 0.1* Beta (SE)=-3.09 (10.92), p not less than 0.1† Beta (SE)=-8.83 (11.32), p not less than 0.1‡ |  |
|  |  |  | 3,777 (models 1-3) 3,776 (model 4) | 20.6% | Net weekly earnings if employed (55 years) | Not reported | Not reported | Beta (SE)=11.75 (30.82), p not less than 0.1 Beta (SE)=14.46 (29.34), p not less than 0.1* Beta (SE)=5.97 (20.66), p not less than 0.1† Beta (SE)=20.83 (22.68), p not less than 0.1‡ |  |
| Child maltreatment | Cape Area Panel Study | Zheng et al. (2018) | All: 2,644 Male: 1,197 Female: 1,477 | All: 59% Male: 57% Female: 60% | Typical monthly wages (in South African Rands) | Not reported | Not reported | All: Coefficient (SE)=-0.12 (0.04), p<0.01 Males: Coefficient (SE)=-0.06 (0.05), p>0.1 Females: Coefficient (SE)=-0.19 (0.06), p<0.01 | Gender, race, age, age squared, education level, marital status, home language, household size, female-headed household, mother’s education and household per capita income, have a child, in school |
| Child abuse | N/A | Lund et al. (2013) | 3,058 | 2.8% | Labour market participation | 41.9% | Female: OR (95% CI)=4.89 (2.79-8.57)  Male: OR (95% CI)=2.84 (1.39-5.81) | Female: OR (95% CI)=4.92 (2.79-8.65) Male: OR (95% CI)=2.74 (1.33-5.63) Female: OR (95% CI)=5.40 (2.78-10.49)*  Male: OR (95% CI)=1.72 (0.68-4.33)* Female: OR (95% CI)= 5.10 (2.60-9.97) † Male: OR (95% CI)=1.75 (0.69-4.41)† | SES §Additionally adjusted for school grades, educational plans, vocational expectations †Additionally adjusted for school grades, educational plans, vocational expectations, self-rated health |
| Family physical violence (to participant or other family member) | Simmons Longitudinal Study | Paradis et al. (2009) | 346 | 15.9% | Unemployment | 15.2% | OR (95% CI)=1.6 (0.7-3.7), p>0.05 | OR (95% CI)=1.8 (0.8-4.2), p>0.05 | Sex, family adversity, and family history of disorder |
|  |  |  |  | 15.9% | Lack of long-range career goals | 8.4% | OR (95% CI)=0.4 (0.1-1.4), p>0.05 | OR (95% CI)=0.4 (0.1-2.0), p>0.05 |  |
| Physical/sexual abuse | Environmental Risk (E-Risk) Longitudinal Twin Study | Jaffee (2018) | 2,066 | 21.1% | NEET status | 18.0% | OR (95% CI)=2.01 (1.45-2.79), no p value | OR (95% CI)=1.46 (1.04-2.05) OR (95% CI)=1.50(1.06-2.12)* OR (95% CI=1.36 (0.95, 1.94)† OR (95% CI)=1.44 (1.00, 2.08)‡ | Sex, SES, youth IQ *Additionally adjusted for maternal depression, parental antisocial personality, parental substance problems †Additionally adjusted for maternal depression, parental antisocial personality, parental substance problems, count youth mental health problems ‡Additionally adjusted for maternal depression, parental antisocial personality, parental substance problems, count youth mental health problems, adult involvement, neighbourhood poverty |
| Physical/sexual abuse | National Child Development Study (NCDS), the 1958 British birth cohort | Fahy et al. (2017) | 9,137 | 6.3% | Labour participation: part-time work | Not reported | Not reported | RR (95% CI)=0.76 (0.57-1.00), p≥0.05 | Gender and childhood mental health |
|  |  |  |  |  | Labour participation: unemployed | Not reported | Not reported | RR (95% CI)=1.70 (1.05-2.77), p<0.05 |  |
|  |  |  |  |  | Labour participation: permanent sick | Not reported | Not reported | RR (95% CI)=2.41 (1.71-3.39), p<0.05 |  |
|  |  |  |  |  | Labour participation: retired | Not reported | Not reported | RR (95% CI)=1.03 (0.60-1.76), p≥0.05 |  |
|  |  |  |  |  | Labour participation: homemaker/other | Not reported | Not reported | RR (95% CI)=1.11 (0.78-1.58), p≥0.05 |  |
| Physical/sexual abuse | Quebec Longitudinal Study of Kindergarten Children (QLSKC) | Domond et al. (2023) | 1,690 | Physical abuse only: 20.4%  Sexual abuse only: 12.2%  Physical and sexual abuse: 8.3% | Received welfare benefits 1-2 years | Physical abuse only: received welfare 1-2 years, 9.1%, ≥5 years, 11.0%  Sexual abuse only: received welfare 1-2 years, 11.0%, ≥5 years, 10.0%  Physical and sexual abuse: received welfare 1-2 years, 15.7.0%, ≥5 years, 13.8% | Physical abuse only: RR (95% CI)=2.68 (1.86-3.86)  Sexual abuse only: RR (95% CI)=2.33 (1.53-3.54)  Physical and sexual abuse: RR (95% CI)=3.60 (2.33-5.55) | Physical abuse only: RR (95% CI)=2.27 (1.51-3.39)  Sexual abuse only: RR (95% CI)=1.51 (0.95-2.42)  Physical and sexual abuse: RR (95% CI)=1.97 (1.23-3.15) | Child sex, child IQ, disruptive sample membership, maternal age at childbirth, paternal age at childbirth, family structure, maternal and paternal education, parental income, mother's employment status during child's preschool years, concurrent mental health problems at 22 years |
| Physical/emotional abuse | Taiwan Youth Project Surveys | Lin and Chiao (2021) | All: 2,897 Male: 1,433 Female: 1,464 | Not reported | NEET | Not reported | Not reported | All: OR (SE)=1.29 (0.27), p not less than 0.05 Males: OR (SE)=1.11 (0.29), p not less than 0.1 Females: OR (SE)=1.82 (0.61), p<0.1 | Sex, school year at enrolment, county, education level, only child, individual covariates (early pubertal maturation, lack of sleep, substance use, educational aspiration, class rank, depressive symptoms, self-esteem), family covariates (parental control, family cohesion) |
| Any child maltreatment (no parental DVA) | Avon Longitudinal Study of Parents and Children (ALSPAC) | Herbert et al. (2023) | 6,252 | Male: 29.5%  Female: 29.9% | Mean earnings loss | Mean earnings=£1,578.18 | Not reported | Coefficient (95%CI)=96.89 (152.92, 40.85), p≤0.001 | Ethnicity, sexual orientation, deprivation subject to IPV as young person, maternal qualifications |
| Any child maltreatment and/or parental DVA | Avon Longitudinal Study of Parents and Children (ALSPAC) | Herbert et al. (2023) | 6,252 | Male: 49.0%  Female: 49.1% | Mean earnings loss | Mean earnings=£1,574.60 | Not reported | Coefficient (95%CI)=88.77, (137.96, 39.57), p≤0.001 | Ethnicity, sexual orientation, deprivation subject to IPV as young person, maternal qualifications |
| Any child maltreatment and/or parental DVA (child aware) | Avon Longitudinal Study of Parents and Children (ALSPAC) | Herbert et al. (2023) | 6,252 | Male: 43.2%  Female: 43.3% | Mean earnings loss | Mean earnings=£1,581.26 | Not reported | Coefficient (95%CI)=106.27 (156.09, 56.46), p≤0.001 | Ethnicity, sexual orientation, deprivation subject to IPV as young person, maternal qualifications |
| Emotional abuse and parental DVA | Avon Longitudinal Study of Parents and Children (ALSPAC) | Herbert et al. (2023) | 6,252 | Male:: 7.9%  Female: 7.7% | Mean earnings loss | Mean earnings=£1,548.14 | Not reported | Coefficient (95%CI)=134.79 (231.49, 38.09), p≤0.01 | Ethnicity, sexual orientation, deprivation subject to IPV as young person, maternal qualifications |
| Emotional abuse and physical abuse and parental DVA | Avon Longitudinal Study of Parents and Children (ALSPAC) | Herbert et al. (2023) | 6,252 | Male: 4.2%  Female: 4.7% | Mean earnings loss | Mean earnings=£1,535.83 | Not reported | Coefficient (95%CI)=222.92 (354.44, 91.39), p≤0.001 | Ethnicity, sexual orientation, deprivation subject to IPV as young person, maternal qualifications |
| Any child maltreatment AND parental DVA | Avon Longitudinal Study of Parents and Children (ALSPAC) | Herbert et al. (2023) | 6,252 | Male: 12.5%  Female: 11.6% | Mean earnings loss | Mean earnings=£1,578.18 | Not reported | Coefficient (95%CI)=121.59 (203.03, 40.15), p≤0.01 | Ethnicity, sexual orientation, deprivation subject to IPV as young person, maternal qualifications |
| Any child maltreatment AND parental DVA (child aware) | Avon Longitudinal Study of Parents and Children (ALSPAC) | Herbert et al. (2023) | 6,252 | Male: 1.2%  Female: 1.8% | Mean earnings loss | Mean earnings=£1,577.65 | Not reported | Coefficient (95%CI)=333.87 (479.52, 188.23), p≤0.001 | Ethnicity, sexual orientation, deprivation subject to IPV as young person, maternal qualifications |
| ***Polyvictimisation*** | | | | | | | | | |
| Polyvictimisation (includes physical violence, sexual abuse, being bullied) | The Youth Studies | Strom et al. (2013) | 11,522 | Physical violence: 13.5% Sexual abuse: 18.7% Being bullied: 8.3% Physical violence and bullying: 4.5% Sexual abuse and bullying: 0.4% Physical violence and sexual abuse: 1.1% Physical violence, sexual abuse and bullying: 0.5% | Lower work participation | Physical violence: 21.4% Sexual abuse: 18.6% Being bullied: 24.0% Physical violence and bullying: 72.7% Sexual abuse and bullying: 75.6% Physical violence and sexual abuse: 76.0% Physical violence, sexual abuse and bullying: 70.2% | OR (95% CI)=1.46 (1.28–1.67), p<0.001 OR (95% CI)=1.23 (0.87-1.74), p>0.05 OR (95% CI)=1.74 (1.48–2.04), p<0.001 OR (95% CI)=1.20 (1.63-2.44), p<0.001 OR (95% CI)=1.78 (0.91-3.49), p>0.05 OR (95% CI)=1.83 (1.22–2.75), p<0.001 OR (95% CI)=0.28 (1.30-3.99), p<0.001 | OR (95% CI)=1.39 (1.19–1.60), p<0.001 OR (95% CI)=1.20 (0.84-1.73), p>0.05 OR (95% CI)=1.61 (1.36–1.91), p<0.001 OR (95% CI)=1.73 (1.39-2.14), p<0.001 OR (95% CI)=1.35 (0.67-2.73), p>0.05 OR (95% CI)=1.71 (1.11–2.62), p<0.05 OR (95% CI)=1.39 (0.73-2.64), p>0.05 | Gender, age, economic situation, parents’ education, parents’ birthplace, parents’ marital situation, father’s employment, mother’s employment, and living situation. |
| Non-sexual abuse (includes physical abuse, psychological abuse and witnessing IPV) | National Child Development Study (NCDS), the 1958 British birth cohort | Pereira et al. (2016) | 8,076 | Score of 1: 7.9% Score of 2-3: 5.5% | Long-term sickness absence | Not reported | Score of 1: OR (95% CI)=2.06 (1.47-2.89)^§^ Score of 2-3: OR (95% CI)=2.77 (1.93-3.98)^§^ | Score of 1: OR (95% CI)=1.75 (1.22-2.51) Score of 2-3: OR (95% CI)=2.10 (1.40-3.17) | Sex, maternal age, birth weight, birth order, poor childhood health, social class in 1958, parental education, household amenities, crowding, and tenure |
|  |  |  |  |  | NEET status | Not reported | Score of 1: OR (95% CI)=1.33 (0.99-1.79)^§^ Score of 2-3: OR (95% CI)=1.62 (1.17-2.24)^§^ | Score of 1: OR (95% CI)=1.20 (0.89–1.63) Score of 2-3: OR (95% CI)=1.33 (0.93-1.90) |  |
|  |  |  |  |  | Income-related support | Not reported | Score of 1: OR (95% CI)=1.34 (1.08-1.67)^§^ Score of 2-3: OR (95% CI)=1.49 (1.16-1.91)^§^ | Score of 1: OR (95% CI)=1.29 (1.03-1.61) Score of 2-3: OR (95% CI)=1.32 (1.00-1.74) |  |
|  |  |  |  |  | Manual occupation^¶^ | Not reported | Score of 1: OR (95% CI)=0.97 (0.79-1.18)^§^ Score of 2-3: OR (95% CI)=1.15 (0.90-1.45)^§^ | Score of 1: OR (95% CI)=0.79 (0.64-0.98) Score of 2-3: OR (95% CI)=0.82 (0.63-1.07) |  |
|  |  |  |  |  | Intergenerational social mobility (birth to 50 years) | Not reported | Not reported | Score of 1: Upward vs. stable manual: OR (95% CI)=1.24 (0.98-1.57) Score of 1: Downward vs. stable nonmanual: OR (95% CI)=0.93 (0.59-1.48) Score of 2-3: Upward vs stable manual: OR (95% CI)=1.14 (0.86-1.53) Score of 2-3: Downward vs stable manual: OR (95% CI)=0.95 (0.53-1.70) | Sex, other types of child maltreatment |
|  |  |  |  |  | Intragenerational social mobility (23 to 50 years) | Not reported | Not reported | Score of 1: Upward vs. stable manual: OR (95% CI)=1.46 (1.08-1.98) Score of 1: Downward vs. stable nonmanual: OR (95% CI)=1.11 (0.81-1.53) Score of 2-3: Upward vs. stable manual: OR (95% CI)=1.30 (0.87–1.94) Score of 2-3: Downward vs. stable nonmanual: OR (95% CI)=1.15 (0.76–1.74) |  |
| Number of maltreatment types (includes non-sexual abuse, neglect and emotional neglect) | National Child Development Study (NCDS), the 1958 British birth cohort | Pereira et al. (2016) | 8,076 | 1 maltreatment type: 20.6% 2+ maltreatment types: 10.3% | Long-term sickness absence | Not reported | Per additional maltreatment experienced (0, 1 and ≥2): OR (95% CI)=1.49 (1.36-1.63) | Per additional maltreatment experienced (0, 1 and ≥2): OR (95% CI)=1.65 (1.42-1.93) | Sex, maternal age, birth weight, birth order, poor childhood health, social class in 1958, parental education, household amenities, crowding, and tenure |
|  |  |  |  |  | NEET status | Not reported | Per additional maltreatment experienced (0, 1 and ≥2): OR (95% CI)=1.26 (1.16-1.37) | Per additional maltreatment experienced (0, 1 and ≥2): OR (95% CI)=1.29 (1.13-1.47) |  |
|  |  |  |  |  | Income-related support | Not reported | Per additional maltreatment experienced (0, 1 and ≥2): OR (95% CI)=1.15 (1.08-1.22) | Per additional maltreatment experienced (0, 1 and ≥2): OR (95% CI)=1.16 (1.06-1.27) |  |
|  |  |  |  |  | Manual occupation^¶^ | Not reported | Per additional maltreatment experienced (0, 1 and ≥2): OR (95% CI)=1.28 (1.21-1.35) | Per additional maltreatment experienced (0, 1 and ≥2): OR (95% CI)=1.13 (1.07-1.21) |  |
| We presented the most adjusted analysis from the papers except for when there were additional adjustments which included something that may be on the causal pathway when we then used the most adjusted analysis without this  ^§^Crude estimates are sex-adjusted  *, †, ‡ symbols represent models with different level of adjustment. The variables included in each model are denoted by the corresponding symbols in the final column of the same row.  ^¶^These outcomes were described by authors using slightly different terminology, but we considered them to be measuring manual occupation | | | | | | | | | |
