# Supplementary material for: How Does Experience of Violence in Childhood Affect Work Outcomes? A Systematic Review of Longitudinal Studies
Source: Trauma Violence Abuse. 2025 Nov 28;27(5):2085–109. doi: 10.1177/15248380251376352 (PMC13736292; doi:10.1177/15248380251376352)
Supplement: Supplementary file 4 [file sj-docx-4-tva-10.1177_15248380251376352.docx]

**Appendix 4: Results for mediation analyses of the relationship between violence against children and work outcomes**

| **Exposure** | **Mediators assessed** | **Outcome** | **Mediation analysis result** | **Authors, year** |
| --- | --- | --- | --- | --- |
| Physical violence | Completion of high school within 5 years | Work participation | *Without mediator*: OR (95% CI)=1.39 (1.19–1.60), p<0.001 *With mediator*: OR (95% CI)=1.19 (1.03-1.38), p<0.05 | Strom et al., 2013 |
| Physical beating | Educational attainment and occupational status | Income deficits | *Without mediator:* Unstandardised coefficient(SE)= -1.13 (0.36), p<0.001  *With mediator:* Unstandardised coefficient(SE)= -0.22 (0.31), p not less than 0.05 | Macmillan (2000) |
| Attacked with weapon | Educational attainment and occupational status | Income deficits | *Without mediator:* Unstandardised coefficent(SE)= -0.69 (0.33), p<0.001 *With mediator:* Unstandardised coefficient(SE)= -0.56 (0.34), p<0.05 | Macmillan, 2000 |
| Sexual assault | Educational attainment and occupational status | Income deficits | *Without mediator:* Unstandardised coefficent(SE)= -0.45 (0.33), p>0.05  *With mediator:* Unstandardised coefficient(SE)= -0.13(0.31), p not less than 0.05 | Macmillan, 2000 |
| Sexual abuse | Mental health | Income-related support | *Without mediator:* OR (95%CI)=2.06 (1.35–3.14) *With mediator:* OR (95%CI)=1.04 (0.52-2.06) | Pereira et al., 2016 |
| Sexual abuse | Cognition | Income-related support | *Without mediator:* OR (95% CI)=2.06 (1.35–3.14) *With mediator:* OR (95% CI)=1.05 (0.54-2.06) | Pereira et al., 2016 |
| Sexual abuse | Mental health Cognition | Income-related support | *Without mediator:* OR (95% CI)=2.06 (1.35–3.14) *With mediator:* OR (95% CI)=1.01 (0.51-1.99) | Pereira et al., 2016 |
| Sexual abuse | Completion of high school within 5 years | Work participation | *Without mediator:* OR (95% CI)=1.20 (0.84–1.73), p>0.05 *With mediator:* OR (95% CI)=1.02 (0.70-1.47), p>0.05 | Strom et al., 2013 |
| Sexual abuse | Mental health | Manual social class | *Without mediator:* OR (95% CI)=1.73 (1.08–2.76) *With mediator:* OR (95% CI)=6.30 (2.62-15.13) | Pereira et al., 2016 |
| Sexual abuse | Cognition | Manual social class | *Without mediator:* OR (95% CI)=1.73 (1.08–2.76) *With mediator:* OR (95% CI)=5.19 (2.14-12.57) | Pereira et al., 2016 |
| Sexual abuse | Mental health Cognition | Manual social class | *Without mediator:* OR (95% CI)=1.73 (1.08–2.76) *With mediator:* OR (95% CI)=5.49 (2.26-13.30) | Pereira et al., 2016 |
| Bullying | Partnership status Highest educational qualification Psychological distress (At age 33) | Economic status | *Without mediator:* Female: Occasionally bullied: 1.34 (0.97, 1.84); p<0.1 Female: Frequently bullied: 1.39 (0.94, 2.06); p<0.1 Male: Frequently bullied: 1.49 (1.04, 2.13); p<0.05  *With mediator:* Female: Occasionally bullied: OR=1.07 (0.73, 1.56); p≥0.1 Female: Frequently bullied: OR=1.22 (0.78, 1.91); p≥0.1 Male: Frequently bullied: OR=1.37 (0.92, 2.06); p≥0.1 | Brimblecombe et al., 2018 |
| Bullying | Partnership status Highest educational qualification Psychological distress (At age 33) | Earnings | *Without mediator:* Frequently bullied: Mean difference= −22.74 (−42.05, −3.43), p<0.1  *With mediator:* Frequently bullied: Mean difference= −16.58 (-55.22, 2.12); p<0.1  (Not analysed for being occasionally bullied or in males or as no significant effect) | Brimblecombe et al., 2018 |
| Bullying | Completion of high school within 5 years | Work participation | *Without mediator:* OR (95% CI)=1.51 (1.27–1.79), p<0.001 *With mediator:* OR (95% CI)=1.51 (1.27-1.79), p<0.001 | Strom et al., 2013 |
| Adolescent IPV | Educational attainment | Earnings | b=-342.81, p<0.01 | Adams et al., 2013 |
| Adolescent IPV | Educational attainment | Growth in earnings | b=-441.56, p<0.01 | Adams et al., 2013 |
| Emotional/physical IPV | Reciprocal IPV (Ages 18-25) | Current employment status | No significant effect | Heng, 2015 |
| Emotional/physical IPV | IPV perpetration (Ages 18-25) | Current employment status | No significant effect | Heng, 2015 |
| Emotional/physical IPV | Depression (Ages 18-25) | Current employment status | Indirect effect= -0.03, p=0.03, 95% CI (-0.07, -0.01) | Heng, 2015 |
| Neglect | Mental health | NEET | *Without mediator:* OR (95% CI)=1.47 (1.13–1.90) *With mediator:* OR (95% CI)=1.50 (1.05-2.15) | Pereira et al., 2016 |
| Neglect | Cognition | NEET | *Without mediator*: OR (95% CI)=1.47 (1.13–1.90) *With mediator:* OR (95% CI)=1.39 (0.93-2.06) | Pereira et al., 2016 |
| Neglect | Cognition Mental health | NEET | *Without mediator:* OR (95% CI)=1.47 (1.13–1.90) *With mediator:* OR (95% CI)=1.41 (0.95-2.09) | Pereira et al., 2016 |
| Neglect | Mental health | Long-term sickness absence | *Without mediator:* OR (95% CI)=1.77 (1.34-2.33) *With mediator:* OR (95% CI)=1.53 (1.06-2.20) | Pereira et al., 2016 |
| Neglect | Cognition | Long-term sickness absence | *Without mediator:* OR (95% CI)=1.77 (1.34-2.33) *With mediator:* OR (95% CI)=1.32 (0.88-1.99) | Pereira et al., 2016 |
| Neglect | Mental health Cognition | Long-term sickness absence | *Without mediator:* OR (95% CI)=1.77 (1.34-2.33) *With mediator:* OR (95% CI)=1.30 (0.85-1.98) | Pereira et al., 2016 |
| Neglect | Mental health | Manual social class | *Without mediator:* OR (95% CI)=1.78 (1.53–2.06) *With mediator:* OR (95% CI)=1.71 (1.42-2.07) | Pereira et al., 2016 |
| Neglect | Cognition | Manual social class | *Without mediator:* OR (95% CI)=1.78 (1.53–2.06) *With mediator:* OR (95% CI)=1.28 (1.04-1.59) | Pereira et al., 2016 |
| Neglect | Mental health Cognition | Manual social class | *Without mediator:* OR (95% CI)=1.78 (1.53–2.06) *With mediator:* OR (95% CI)=1.24 (1.01-1.54) | Pereira et al., 2016 |
| Physical neglect | Education | Earnings | p<0.01 (explains 60% of association) | Schurer et al. 2019 |
| Physical neglect | Labour force attachment | Earnings | p<0.01 | Schurer et al. 2019 |
| Physical neglect | Cognition | Earnings | p<0.01 (explains 23% of association) | Schurer et al. 2019 |
| Physical neglect | Non-cognitive skills | Earnings | p<0.05 (explains 8% of association) | Schurer et al. 2019 |
| Physical neglect | Health | Earnings | Not significant | Schurer et al. 2019 |
| Physical neglect | Family factors (marital status, no. children) | Earnings | Not significant | Schurer et al. 2019 |
| Physical neglect | Partnership status | Earnings | Not significant | Schurer et al. 2019 |
| Physical neglect | Education | Welfare dependence | p<0.01 | Schurer et al. 2019 |
| Physical neglect | Health | Welfare dependence | Not significant | Schurer et al. 2019 |
| Physical neglect | Family factors (marital status, no. children) | Welfare dependence | Not significant | Schurer et al. 2019 |
| Physical neglect | Partnership status | Welfare dependence | Not significant | Schurer et al. 2019 |
| Physical neglect | Labour force attachment | Welfare dependence | Not significant | Schurer et al. 2019 |
| Physical neglect | Cognition | Welfare dependence | p<0.05 | Schurer et al. 2019 |
| Physical neglect | Non-cognitive skills | Welfare dependence | p<0.01 | Schurer et al. 2019 |
| Adolescent victimisation | Educational attainment and occupational status | Income deficits | Without mediator: Unstandardised coefficient(SE)= -1.03 (0.27), p<0.001  *With mediator:* Unstandardised coefficient (SE)= -0.52 (0.26), p<0.001 | Macmillan, 2000 |
| Victimisation | Educational investment Educational effort Grade point average Total educational attainment | Occupational status | Beta=-0.05 (significant indirect effect) | Macmillan, 1998 |
| Childhood victimisation | Years of education Student enrolment status | Occupational prestige | Years of education (*indirect*): Beta (SE)=-0.84 (0.05), p<0.001 Student enrolment (*indirect*): Beta (SE)=0.10 (0.01), p<0.001 *Total effect* (*indirect + direct*, both mediators): -0.62 (0.10), p<0.001 | Fernandez et al., 2015 |
| Childhood victimisation | Years of education Student enrolment status | Annual income | Years of education (*indirect*): Beta (SE)= $-1,036 ($71), p<0.001 Student enrolment (*indirect*): Beta (SE)=$448 ($40), p<0.001 *Total effect* (*indirect + direct*, both mediators): -$891 ($153), p<0.001 | Fernandez et al., 2015 |
| Non-sexual abuse | Mental health | Income-related support | *Without mediator:* 1 vs 0: OR (95% CI)=1.29 (1.03–1.61) 2-3 vs 0: OR (95% CI)=1.32 (1.00–1.74)  *With mediator:*  1 vs 0: OR (95% CI)=1.24 (0.95–1.63) 2-3 vs 0: OR (95% CI)=1.33 (0.92–1.91) | Pereira et al., 2016 |
| Non-sexual abuse | Cognition | Income-related support | *Without mediator:* 1 vs 0: OR (95% CI)=1.29 (1.03–1.61) 2-3 vs 0: OR (95% CI)=1.32 (1.00–1.74)  *With mediator:* 1 vs 0: OR (95% CI)=1.29 (0.99–1.70) 2-3 vs 0: OR (95% CI)=1.50 (1.05–2.15) | Pereira et al., 2016 |
| Non-sexual abuse | Mental health Cognition | Income-related support | *Without mediator:* 1 vs 0: OR (95% CI)=1.29 (1.03–1.61) 2-3 vs 0: OR (95% CI)=1.32 (1.00–1.74)  *With mediator:* 1 vs 0: OR (95% CI)=1.28 (0.98–1.68) 2-3 vs 0: OR (95% CI)=1.39 (0.96–2.01) | Pereira et al., 2016 |
| Non-sexual abuse | Mental health | Long-term sickness absence | *Without mediator:* 1 vs 0: OR (95% CI)=1.75 (1.22–2.51) 2-3 vs 0: OR (95% CI)=2.10 (1.40–3.17)  *With mediator:* 1 vs 0: OR (95% CI)= 2.11 (1.42–3.13) 2-3 vs 0: OR (95% CI)=2.32 (1.34–4.01) | Pereira et al., 2016 |
| Non-sexual abuse | Cognition | Long-term sickness absence | *Without mediator:* 1 vs 0: OR (95% CI)=1.75 (1.22–2.51) 2-3 vs 0: OR (95% CI)=2.10 (1.40–3.17)  *With mediator:* 1 vs 0: OR (95% CI)=2.27 (1.52-3.38) 2-3 vs 0: OR (95% CI)=2.56 (1.51-4.34) | Pereira et al., 2016 |
| Non-sexual abuse | Mental health Cognition | Long-term sickness absence | *Without mediator:* 1 vs 0: OR (95% CI)=1.75 (1.22–2.51) 2-3 vs 0: OR (95% CI)=2.10 (1.40–3.17)  *With mediator*: 1 vs 0: OR (95% CI)=2.22 (1.49–3.31) 2-3 vs 0: OR (95% CI)=2.43 (1.38–4.26) | Pereira et al., 2016 |
| Physical violence and bullying | Completion of high school within 5 years | Work participation | *Without mediator:* OR (95% CI)=1.73 (1.39–2.14), p<0.001 *With mediator:* OR (95% CI)=1.51 (1.21-1.88), p<0.001 | Strom et al., 2013 |
| Sexual abuse and physical violence | Completion of high school within 5 years | Work participation | *Without mediator:* OR (95% CI)=1.71 (1.11–2.62), p<0.05 *With mediator:* OR (95% CI)=1.18 (0.57-2.43), p>0.05 | Strom et al., 2013 |
| Sexual abuse and bullying | Completion of high school within 5 years | Work participation | *Without mediator:* OR (95% CI)=1.35 (0.67–2.73), p>0.05 *With mediator:* OR (95% CI)=1.18 (0.57–2.43), p>0.05 | Strom et al., 2013 |
| Sexual abuse, physical violence and bullying | Completion of high school within 5 years | Work participation | *Without mediator:* OR (95% CI)=0.39 (0.73–2.64), p>0.05 *With mediator:* OR (95% CI)=0.89 (0.46–1.71) | Strom et al., 2013 |
